# On the sources and sinks of atmospheric VOCs: an integrated analysis of recent aircraft campaigns over North America

**DOI:** 10.5194/acp-19-9097-2019

**Published:** 2019-07-17

**Authors:** Xin Chen, Dylan B. Millet, Hanwant B. Singh, Armin Wisthaler, Eric C. Apel, Elliot L. Atlas, Donald R. Blake, Ilann Bourgeois, Steven S. Brown, John D. Crounse, Joost A. de Gouw, Frank M. Flocke, Alan Fried, Brian G. Heikes, Rebecca S. Hornbrook, Tomas Mikoviny, Kyung-Eun Min, Markus Müller, J. Andrew Neuman, Daniel W. O'Sullivan, Jeff Peischl, Gabriele G. Pfister, Dirk Richter, James M. Roberts, Thomas B. Ryerson, Stephen R. Shertz, Chelsea R. Thompson, Victoria Treadaway, Patrick R. Veres, James Walega, Carsten Warneke, Rebecca A. Washenfelder, Petter Weibring, Bin Yuan

**Affiliations:** 1 Department of Soil, Water, and Climate, University of Minnesota, Minneapolis-Saint Paul, MN, USA; 2 NASA Ames Research Center, Moffett Field, CA, USA; 3 Institute for Ion Physics and Applied Physics, University of Innsbruck, 6020 Innsbruck, Austria; 4 Department of Chemistry, University of Oslo, Oslo, Norway; 5 Atmospheric Chemistry Observations & Modeling Laboratory, National Center for Atmospheric Research, Boulder, CO, USA; 6 Department of Atmospheric Sciences, Rosenstiel School of Marine and Atmospheric Science, University of Miami, Miami, FL, USA; 7 Department of Chemistry, University of California, Irvine, Irvine, CA, USA; 8 Chemical Sciences Division, NOAA Earth System Research Laboratory, Boulder, CO, USA; 9 Cooperative Institute for Research in Environmental Sciences, University of Colorado, Boulder, CO, USA; 10 Division of Geological and Planetary Sciences, California Institute of Technology, Pasadena, CA, USA; 11 Institute of Arctic & Alpine Research, University of Colorado, Boulder, CO, USA; 12 Graduate School of Oceanography, University of Rhode Island, Narragansett, RI, USA; 13 School of Earth Science and Environmental Engineering, Gwangju Institute of Science and Technology, Gwangju, South Korea; 14 United States Naval Academy, Chemistry Department, Annapolis, MD, USA; 15 Institute for Environmental and Climate Research, Jinan University, Guangzhou, China

## Abstract

We apply a high-resolution chemical transport model (GEOS-Chem CTM) with updated treatment of volatile organic compounds (VOCs) and a comprehensive suite of airborne datasets over North America to (i) characterize the VOC budget and (ii) test the ability of current models to capture the distribution and reactivity of atmospheric VOCs over this region. Biogenic emissions dominate the North American VOC budget in the model, accounting for 70 % and 95 % of annually emitted VOC carbon and reactivity, respectively. Based on current inventories anthropogenic emissions have declined to the point where biogenic emissions are the dominant summertime source of VOC reactivity even in most major North American cities. Methane oxidation is a 2x larger source of nonmethane VOCs (via production of formaldehyde and methyl hydroperoxide) over North America in the model than are anthropogenic emissions. However, anthropogenic VOCs account for over half of the ambient VOC loading over the majority of the region owing to their longer aggregate lifetime. Fires can be a significant VOC source episodically but are small on average. In the planetary boundary layer (PBL), the model exhibits skill in capturing observed variability in total VOC abundance (*R*^2^ = 0:36) and reactivity (*R*^2^ = 0:54). The same is not true in the free troposphere (FT), where skill is low and there is a persistent low model bias (~ 60 %), with most (27 of 34) model VOCs underestimated by more than a factor of 2. A comparison of PBL: FT concentration ratios over the southeastern US points to a misrepresentation of PBL ventilation as a contributor to these model FT biases. We also find that a relatively small number of VOCs (acetone, methanol, ethane, acetaldehyde, formaldehyde, isoprene C oxidation products, methyl hydroperoxide) drive a large fraction of total ambient VOC reactivity and associated model biases; research to improve understanding of their budgets is thus warranted. A source tracer analysis suggests a current overestimate of biogenic sources for hydroxyacetone, methyl ethyl ketone and glyoxal, an underestimate of biogenic formic acid sources, and an underestimate of peroxyacetic acid production across biogenic and anthropogenic precursors. Future work to improve model representations of vertical transport and to address the VOC biases discussed are needed to advance predictions of ozone and SOA formation.

## 1 Introduction

Volatile organic compounds (VOCs) play a central role in atmospheric chemistry. Through their influence on the hydroxyl radical (OH), VOCs alter the lifetime of long-lived greenhouse gases (Cubasch et al., 2013), while their oxidation products such as ozone (O_3_) and secondary organic aerosol (SOA) degrade human and ecosystem health (EPA, 2018) and alter Earth’s radiative balance (Myhre et al., 2013). There are large uncertainties associated with the emissions (Karl et al., 2018; Hatch et al., 2017; Guenther et al., 2012), chemical processing (Caravan et al., 2018; Shaw et al., 2018; J. F. Müller et al., 2016), and sinks of atmospheric VOCs (Iavorivska et al., 2017; Nguyen et al., 2015; Wolfe et al., 2015; Karl et al., 2010). An ensemble of recent airborne campaigns over North America together afford the most expansive picture yet of the atmospheric VOC distribution over this region. Here we apply a high-resolution chemical transport model (nested GEOS-Chem CTM) with a new and highly comprehensive VOC treatment to (1) interpret that observational ensemble in terms of their constraints on the distribution, speciation, and sources of VOC carbon and reactivity; (2) assess our current scientific ability to capture that distribution across diverse environments; and (3) identify priorities for future research and model improvements.

It is widely recognized that terrestrial ecosystems provide the largest source of VOCs to the global atmosphere, mainly through foliar emissions but also via microbial decomposition of organic material, with an estimated flux of 750–1000 Tg yr^−1^ (Safieddine et al., 2017; Guenther et al., 2012). Global anthropogenic VOC emissions are thought to be an order of magnitude lower (e.g., 100–160 Tg yr^−1^, Gla-sius and Goldstein, 2016; Boucher et al., 2013), and include contributions from mobile sources such as on-road vehicles and aircraft (Stettler et al., 2011; Parrish, 2006) and from stationary sources such as volatile chemical products, fuel production, distribution, and combustion, and waste treatment (McDonald et al., 2018; Warneke et al., 2014; de Gouw et al., 2012; Millet et al., 2012). Biomass burning, i.e., combustion of any nonfossilized vegetation, leads to an estimated 60–400 Tg yr^−1^ of emitted VOCs, though with high uncertainty regarding potential unidentified and/or unmeasured pyrogenic compounds (Giglio et al., 2013; Akagi et al., 2011; Wiedinmyer et al., 2011; Andreae and Merlet, 2001). Ocean-atmosphere VOC fluxes have been investigated with a range of aircraft- and ship-based observations, remote sensing, and modeling approaches for species including isoprene and monoterpenes, other light hydrocarbons, halogenated species, and oxygenated VOCs such as methanol, acetone, formaldehyde, acetaldehyde, glyoxal, and carboxylic acids (Deventer et al., 2018; Kim et al., 2017; Mungall et al., 2017; Coburn et al., 2014; Yang et al., 2013, 2014a, b; Beale et al., 2011, 2013; Fischer et al., 2012; Luo and Yu, 2010; Millet et al., 2008, 2010; Shaw et al., 2010; Read et al., 2008; Palmer and Shaw, 2005; Williams et al., 2004; Singh et al., 2003; Broadgate et al., 1997; Zhou and Mopper, 1997; Bonsang et al., 1988; Kanakidou et al., 1988). However, the quantitative role of the ocean as a net global VOC source or sink remains uncertain (Carpenter et al., 2012; Read et al., 2012).

While there have been a large number of studies focusing on one or a small subset of VOCs (a recent Web of Science search for articles with topic terms (“volatile organic compound*”) AND (“atmospher*”) returned over 6800 results), there have been few integrated studies examining the overall suite of measured species and our ability to capture that ensemble behavior in current CTMs. In one example, de Gouw et al. (2005) examined the photochemical evolution of organic carbon from urban outflow in the northeastern US and found evidence for unidentified aerosol precursors. Later, Goldstein and Galbally (2007) compiled a rough estimate of the total VOC budget and argued that there is a large pool of uncharacterized organic compounds in the atmosphere. Heald et al. (2008) carried out an integrated assessment of total observed organic carbon based on available measurements to that point, and articulated a need for more routine and comprehensive VOC-carbon measurements, while Safieddine et al. (2017) recently performed the first CTM-based budget analysis of total organic carbon on a global scale.

Recent observational work has benefited from new tools (e.g., high-resolution time-of-flight mass spectrometry) that enable a more thorough and time-resolved characterization of VOC carbon than was previously possible. For instance, new flux measurements have been able for the first time to characterize the two-way surface atmosphere exchange of VOC carbon simultaneously across the entire mass spectrum (Karl et al., 2018; Millet et al., 2018; Park et al., 2013). In addition, recent studies (Isaacman-VanWertz et al., 2018; Hunter et al., 2017) combining a comprehensive suite of online instrumentation have been able to achieve organic carbon closure (to within error) in a forested environment and in a laboratory oxidation experiment, respectively.

The past decade has thus seen major advances in the scientific community’s ability to measure (e.g., Glasius and Goldstein, 2016) as well as model (e.g., Safieddine et al., 2017) atmospheric organic carbon, and in our laboratory-derived understanding of key VOC oxidation pathways (e.g., Praske et al., 2018; Ehn et al., 2014; Crounse et al., 2013; Paulot et al., 2009b). Over the same period, there have been a large number of airborne campaigns over North America that, together, are unprecedented in their chemical and spatial coverage for characterizing VOC distributions over this region. Here, we perform an integrated analysis of these airborne datasets based on a high-resolution chemical transport model (nested GEOS-Chem CTM). The model simulation includes the latest updates related to atmospheric VOCs (Sect. 2) and provides a more comprehensive representation of atmospheric organics than has been available for prior model– measurement evaluations. We apply this updated model with the suite of airborne observations to assess present understanding of the processes driving atmospheric VOCs, identify knowledge gaps, and address priorities for future work. We focus in this paper specifically on nonmethane VOCs; we exclude intermediate, semi-volatile, low-volatility, and extremely low-volatility organic compounds (IVOCs, SVOCs, LVOCs, ELVOCs) because a comparable suite of airborne observations does not exist for these. The Hunter et al. study referenced above found for a ponderosa pine forest that while S/IVOC and E/LVOC species accounted for most of the aerosol-forming material, VOCs dominated the ambient OH reactivity due to nonmethane organics and also provided the majority of the organic carbon mass (Hunter et al., 2017). Likewise, while organic aerosol formation and subsequent deposition is not counted explicitly as a VOC sink in our chemical mechanism, prior work has found this to be only a small fraction (< 4 %) of the gas-phase VOC budget (Safieddine et al., 2017).

## 2 Model description

We use the GEOS-Chem CTM (v10–01; http://geos-chem.org, last access: 3 July 2019) driven by assimilated meteorological fields (Goddard Earth Observation System Forward Processing product, GEOS-FP) from the NASA Goddard Modeling and Assimilation Office (GMAO). Simulations are performed for 2013, the year in which several of the utilized aircraft campaigns took place. The GEOS-FP fields have spatial resolution of 0.25° × 0.3125° and temporal resolution of 3 h for 3-D meteorological parameters and 1 h for surface quantities and mixing depths. The North American simulation used here is conducted within a nested framework (9.75–60° N, 130–60° W; 47 vertical layers) at the native GEOS-FP horizontal resolution (Kim et al., 2015), with time steps of 5 min (transport/convection) and 10 min (emissions/chemistry) (Philip et al., 2016). Dynamic boundary conditions are obtained from a global simulation (4° × 5°) with time steps of 30 min (transport/convection) and 60 min (emissions/chemistry). The Supplement (Figs. S1, S2) shows an evaluation of these boundary conditions based on Atmospheric Tomography Mission (ATom) (Wofsy et al., 2018) ozone observation in the northern Pacific. We use the TP-CORE advection algorithm (Lin and Rood, 1996), convective mass fluxes from the GEOS-FP archive (Wu et al., 2007), and the nonlocal boundary layer mixing scheme described by Lin and McElroy (2010).

A year-long nested model run for 2013 was obtained via 12 parallel month-long simulations. Each of the latter was initialized after a ~ 1-week nested spin-up of regridded concentration fields from a ~ 2-year global spin-up. We find that this procedure is sufficient to achieve a dynamic steady state for oxidant and VOC levels in the model, as species that would require longer spin-up (e.g., methane) are prescribed rather than actively simulated in this mechanism.

### 2.1 Chemistry

The chemical mechanism in this work is based on Millet et al. (2018), with the following modifications. Here we incorporate a more detailed treatment of monoterpene chemistry that is adapted from Fisher et al. (2016), along with updated photo-isomerization yields for acetaldehyde (Millet et al., 2015). Further updates are included for VOC ozonolysis (isoprene, methacrolein, and isoprene hydroxynitrate) (Marais et al., 2016), glyoxal and methyl glyoxal yields from aromatics (Fischer et al., 2014), carboxylic acid production from the hydrolysis of stabilized Criegee intermediates (Millet et al., 2015), and photolysis cross sections for methyl vinyl ketone (MVK) and methacrolein (MACR) nitrates and propanone nitrate (Paulot et al., 2009a). Finally, we apply the carbon mass tracking approach outlined in Safieddine et al. (2017) to ensure carbon closure.

### 2.2 Deposition

Physical VOC sinks in GEOS-Chem include dry deposition following the Wesely (1989) scheme as implemented by Wang et al. (1998), and wet deposition as described by Amos et al. (2012). Wet deposition assumes liquid-phase-only uptake of VOCs (except formic acid and acetic acid) with a retention efficiency of 1 in warm clouds and 0.02 in mixed clouds (Mari et al., 2000). Ice uptake of formic acid and acetic acid is included based on the Langmuir isotherm model (Paulot et al., 2011).

Henry’s law solubility constants (*H* values; required for calculating dry deposition resistances, gas-phase wet deposition, and air-sea fluxes) are computed following Travis et al. (2016) and Nguyen et al. (2015) for nitric acid, hydrogen peroxide, and a suite of isoprene-derived oxygenated VOCs (isoprene hydroxy hydroperoxides, isoprene hydroxynitrate, isoprene epoxides, MVK/MACR nitrates, propanone nitrate, glycolaldehyde, hydroxyacetone). Values for lumped ≥ C4 alkylnitrates and formaldehyde are based on Marais et al. (2016) and Jacob (2000), respectively, while those for benzene, toluene, and xylene (representing lumped C8 aromatics) are taken from Staudinger and Roberts (2001). The lumped xylene species in the model uses the mean *H* value from the corresponding individual C8 compounds (*o*-xylene, *m*-xylene, *p*-xylene, ethylbenzene). For other VOCs we use central literature values based on the Sander (2015) compilation. Carboxylic acids employ an effective *H* value at pH = 7, with lumped ≥ C3 acids using the median reported value for propionic acid (Nirmalakhandan and Speece, 1988).

### 2.3 Emissions

#### 2.3.1 Natural emissions

Biogenic VOC emissions from terrestrial plants are calculated online in GEOS-Chem using the Model of Emissions of Gases and Aerosols from Nature version 2.1 (MEGAN v2.1), implemented into GEOS-Chem as described by Hu et al. (2015).

NO_x_ emissions from microbial processes in soils are estimated as described in Hudman et al. (2012). The annual combined global flux of formic and acetic acids from soils estimated previously by Paulot et al. (2011) corresponds to approximately 10 % of this NO_x_ source, and we therefore prescribe the formic acid and acetic acid soil fluxes as 5 % (each) of the Hudman et al. (2012) molar NO_x_ flux.

Marine hydrocarbon emissions (for alkanes, alkenes, and isoprene) are estimated following Millet et al. (2015) and Paulot et al. (2011). Air-sea fluxes of oxygenated VOCs are calculated following Johnson (2010), Millet et al. (2010, 2008), and Fischer et al. (2012), with assumed fixed seawater concentrations of 15 nM (acetone), 31 nM (methanol), and 6 nM (acetaldehyde) based on compiled cruise measurements (Beale et al., 2011, 2013, 2015; Yang et al., 2013, 2014a, b; Kameyama et al., 2009; Hudson et al., 2007; Marandino et al., 2005; Williams et al., 2004; Zhou and Mopper, 1997).

#### 2.3.2 Anthropogenic emissions

Global anthropogenic VOC emissions in the model are from the Interpolated ACCMIP-RCP 8.5 inventory for the year 2013 (van Vuuren et al., 2011; Lamarque et al., 2010; Ri-ahi et al., 2007) (with a few exceptions; see below). This inventory provides speciated emissions for alkanes, alkenes, alkynes, and aromatics, and unspeciated emissions for alcohols, ≥ C2 aldehydes, ketones, and carboxylic acids. For the latter group, we apply speciation factors for methanol and ethanol (0.5, 0.375, mass basis), acetaldehyde and ≥ C3 aldehydes (0.75, 0.25), and acetone and ≥ C4 ketones (0.75, 0.25) based on prior studies (Wells et al., 2012; Millet et al., 2010). Formic acid and acetic acid together are assumed to account for 75 % by mass of the total ACCMIP carboxylic acid source (these in turn are partitioned with a 1: 2 molar ratio), with ≥ C3 carboxylic acids making up the remaining 25 % (Paulot et al., 2011).

Global anthropogenic and biofuel emissions of ethane and propane are from Xiao et al. (2008). Global formic and acetic acid emissions from animal agriculture are scaled to the corresponding ammonia source (from EDGAR v4.2 agricultural sectors 4C and 4D) following Paulot et al. (2011). We use global biofuel emissions from Yevich and Logan (2003) for emitted oxygenated VOCs not included in ACCMIP-RCP 8.5 (glycolaldehyde, hydroxyacetone, glyoxal, and methyl glyoxal). Aircraft emissions are from the AEIC inventory (Stettler et al., 2011), and global anthropogenic NO_x_, CO, SO_2_, and NH_3_ emissions are from EDGAR v4.2 (European Commission (EC), 2011).

Over North America, emissions of inorganic species and VOCs (except ethane and propane) from anthropogenic, biofuel, and ship sources are overwritten by the hourly EPA/NEI2011 inventory (Travis et al., 2016; EPA, 2015), with annual scale factors applied to account for recent trends (e.g., the nationally aggregated 2011–2013 emission trend factor for VOCs is 0.971). Molar fluxes of formic and acetic acid over North America from these sources are estimated by scaling those of CO by 2.1 × 10^−4^ and 4.2 × 10^−4^, respectively (Paulot et al., 2011).

#### 2.3.3 Biomass burning emissions

Open fire emissions are calculated from monthly burned area and fractional fire type contributions from the fourth version of the Global Fire Emissions Database with small fires (GFED4s) (van der Werf et al., 2017) for our simulation year. We use the GFED-recommended species-specific emission factors (http://www.globalfiredata.org/data.html, last access: 3 July 2019) which are based primarily on Akagi et al. (2011).

## 3 Airborne measurements of VOCs over North America

**Figure 1.**
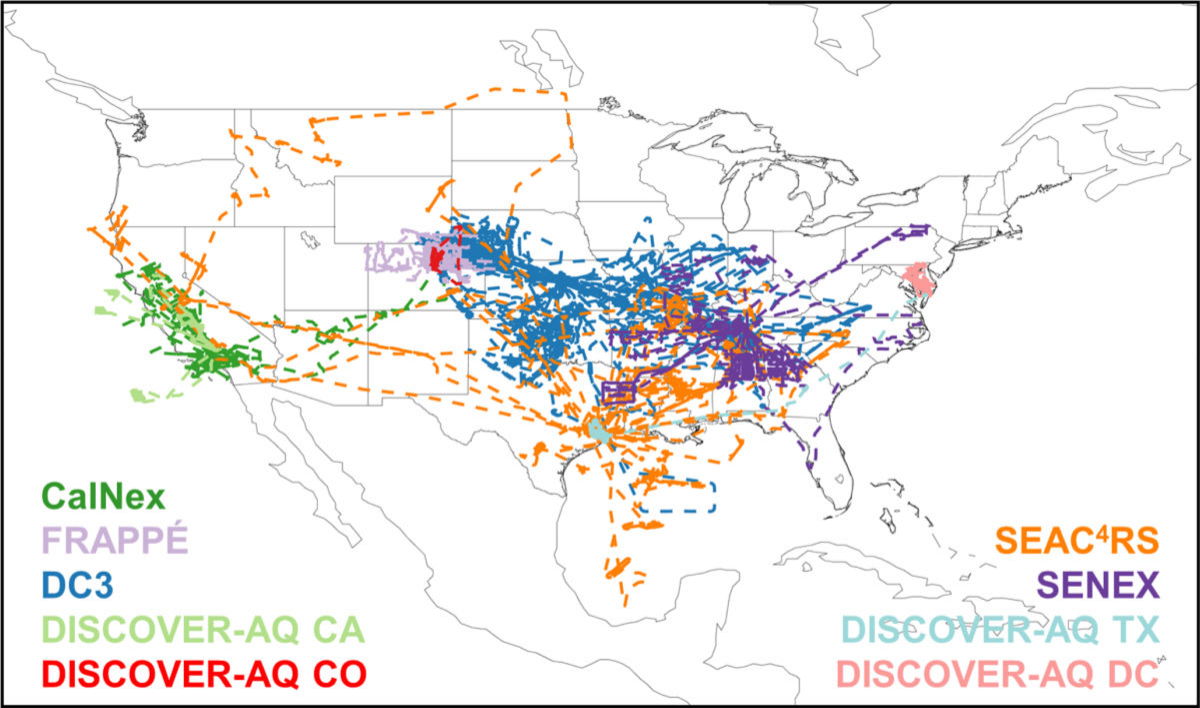
Flight tracks for the aircraft campaigns used in this study: CalNex (May–June 2010), FRAPPÉ (July–August 2014), DC3 (May–June 2012), DISCOVER-AQ CA (January–February 2013), DISCOVER-AQ CO (July–August 2014), SEAC^4^RS (August– September 2013), SENEX (June 2013), DISCOVER-AQ TX (September 2013), and DISCOVER-AQ DC (June–July 2011).

Figure 1 shows flight tracks for the airborne tropospheric chemistry missions that took place over North America between 2010 and 2014 and are used here. We have used data from intensive field campaigns using NCAR, NOAA, and NASA aircraft that carried a large instrument payload to simultaneously measure many VOCs. Together, they provide a rich dataset for constraining VOC-related processes, as they feature extensive horizontal and vertical sampling throughout the North American troposphere and include a range of observing strategies such as survey transects, racetrack gradients/walls, and spirals. Table 1 summarizes the campaigns in terms of sampling time period, region, and aircraft platform and flight ceiling, with instrumental measurement details and references provided in Table S1. Below, we briefly introduce the overall goals and instrument payload for each campaign.

The Studies of Emissions and Atmospheric Composition, Clouds, and Climate Coupling by Regional Surveys (SEAC^4^RS 2013; August–September 2013) (Toon et al., 2016; SEAC^4^RS Science Team, 2013) was conducted over the southeastern US and targeted a broad range of goals including quantifying the regional distribution of anthropogenic, biomass burning, and biogenic chemicals, characterizing their re-distribution through convection, and identifying their impacts on boundary layer and upper tropospheric chemistry. The deployed NASA DC-8 aircraft has a flight ceiling of 12.5 km above sea level (a.s.l.), enabling deep vertical profiling. The SEAC^4^RS VOC payload included a chemical ionization mass spectrometer using CF_3_O^−^ reagent ions (CIT-CIMS (CF3O^−^)), a separate CIMS measuring peroxy acetyl nitrate (PAN-CIMS), a proton-transfer-reaction mass spectrometer (PTR-MS), in situ airborne formaldehyde measurements by laser-induced fluorescence (ISAF-LIF), thermal dissociation LIF (TD-LIF), and a whole air sampler (WAS). Specific VOCs measured by each instrument are listed in Table S1.

The Southeast Nexus (SENEX; June 2013) campaign (Warneke et al., 2016) was part of the Southeast Atmosphere Study (SAS). The NOAA WP-3D aircraft sampled the boundary layer through the mid-troposphere (up to 6.4 km a.s.l.), targeting both natural and anthropogenic emissions. Onboard VOC instruments included WAS, ISAF-LIF, PAN-CIMS, and PTR-MS. SENEX also featured in situ measurements of carboxylic acids by two separate CIMS using iodide reagent ions (I?-CIMS) and of glyoxal via an airborne cavity enhanced spectrometer (ACES) (Table S1).

The Deep Convective Clouds and Chemistry (DC3; May– June 2012) field experiments took place over the central US and were specifically designed to investigate changes in upper tropospheric composition and chemistry during and after deep convective events (Barth et al., 2015; DC3 Science Team, 2013). During DC3 the NASA DC-8 and GV aircraft sampled storm outflow up to 13 km a.s.l. through spirals and wall sampling. The VOC payload included PTR-MS, a Trace Organic Gas Analyzer (TOGA), CIT-CIMS (CF3O^−^), PAN-CIMS, ISAF-LIF, TD-LIF, and WAS.

The California Research at the Nexus of Air Quality and Climate Change (CalNex; May–June 2010) campaign studied air quality and climate over California and offshore (Ryerson et al., 2013). The NOAA WP-3D aircraft sampled the troposphere up to 5 km a.s.l., and carried out survey tracks over the northern, central, and southern San Joaquin Valley and Los Angeles basin, with spirals over targeted urban and agricultural sources. VOCs were measured onboard by PTR-MS, PAN-CIMS, and WAS.

DISCOVER-AQ (Deriving Information on Surface Conditions from Column and Vertically Resolved Observations Relevant to Air Quality) (Crawford and Pickering, 2014; DISCOVER-AQ Science Team, 2014) included four separate airborne campaigns: DISCOVER-AQ DC (June-July 2011) over Baltimore-Washington DC, DISCOVER-AQ CA (January–February 2013) over the San Joaquin Valley, DISCOVER-AQ TX (September 2013) over Houston, and DISCOVER-AQ CO (July–August 2014) over the Denver, Colorado, urban region. The NASA P3-B aircraft (8.5 km a.s.l. ceiling) was employed in each case, with frequent and repeated spirals to characterize the vertical structure of the troposphere. The VOC payload included a difference frequency generation absorption spectrometer (DFGAS) and time-of-flight PTR-MS (PTR-ToF-MS; quadrupole PTR-MS was used for DISCOVER-AQ DC).

FRAPPÉ (Front Range Air Pollution and Photochemistry Experiment; July–August 2014) took place jointly with DISCOVER-AQ CO, with the employed NCAR C-130 aircraft (8 km a.s.l. ceiling) sampling the broader mountain-plain areas over northern Colorado. The VOC payload included PTR-MS, a compact atmospheric multi-species spectrometer CAMS), TOGA, peroxide CIMS (PCIMS), PAN-CIMS, and WAS.

We use 1 min merged data from each campaign to match the frequency at which the GEOS-Chem output is sampled along the aircraft flight tracks. For species co-measured by multiple instruments during the same campaign, we select one measurement primarily based on time response (≤ 1 min sampling rate preferred), while also considering data availability and nominal accuracy. For example, VOCs measured by PTR-MS, TOGA, or CAMS (for ethane) take precedence over contemporaneous WAS observations due to the higher time resolution. The ISAF-LIF, DFGAS, and CAMS instruments are specifically designed for formaldehyde, and we use these observations (rather than WAS, TOGA, or PTRMS) in all cases with the exception of CalNex (where PTR-MS was the only available HCHO measurement). PTR-MS and TOGA measurements during FRAPPÉ are highly correlated but with 5 %–30 % discrepancies across compounds (Fig. S3). We therefore repeated our main analyses using data from each instrument (see Figs. 5–8 and Tables S2–S5) and find that the conclusions are not significantly changed. Similar sensitivity tests are done for formaldehyde, which had concurrent observations during DC3-DC-8 (DFGAS, ISAF-LIF) and during SEAC^4^RS (CAMS, ISAF-LIF), as well as for formic acid, which had concurrent observations during SENEX (NOAA CIMS, UW CIMS) (Fig. S4).

One concern when combining multiple measurements is the differing time resolution between instruments. For example, the WAS systems collect discrete samples separated by up to 10 min, while TOGA collects a 35 s integrated sample on alternate minutes. Many other instruments used here have significantly higher time resolution. To address this issue, when mapping aggregated quantities (i.e., total VOC carbon; Fig. 5), we consider only those data points with complete species coverage (no missing data within a given campaign’s payload). Overall, this yields ~7000 and ~4500 1 min averaged observational data points in the planetary boundary layer (PBL, defined here as <2 km a.g.l.) and free troposphere (FT, >3 km a.g.l.), respectively, distributed over ~900 and ~1700 model grid cells in each case. Finally, to avoid comparing a single modeled value with multiple observations falling into the same model grid box and time step, all measurements and model output are averaged and gridded to unique model grid-box–time-step combinations.

## 4 Simulated VOC budget over North America

### 4.1 Biogenic emissions dominate the VOC budget on a carbon basis

**Figure 2.**
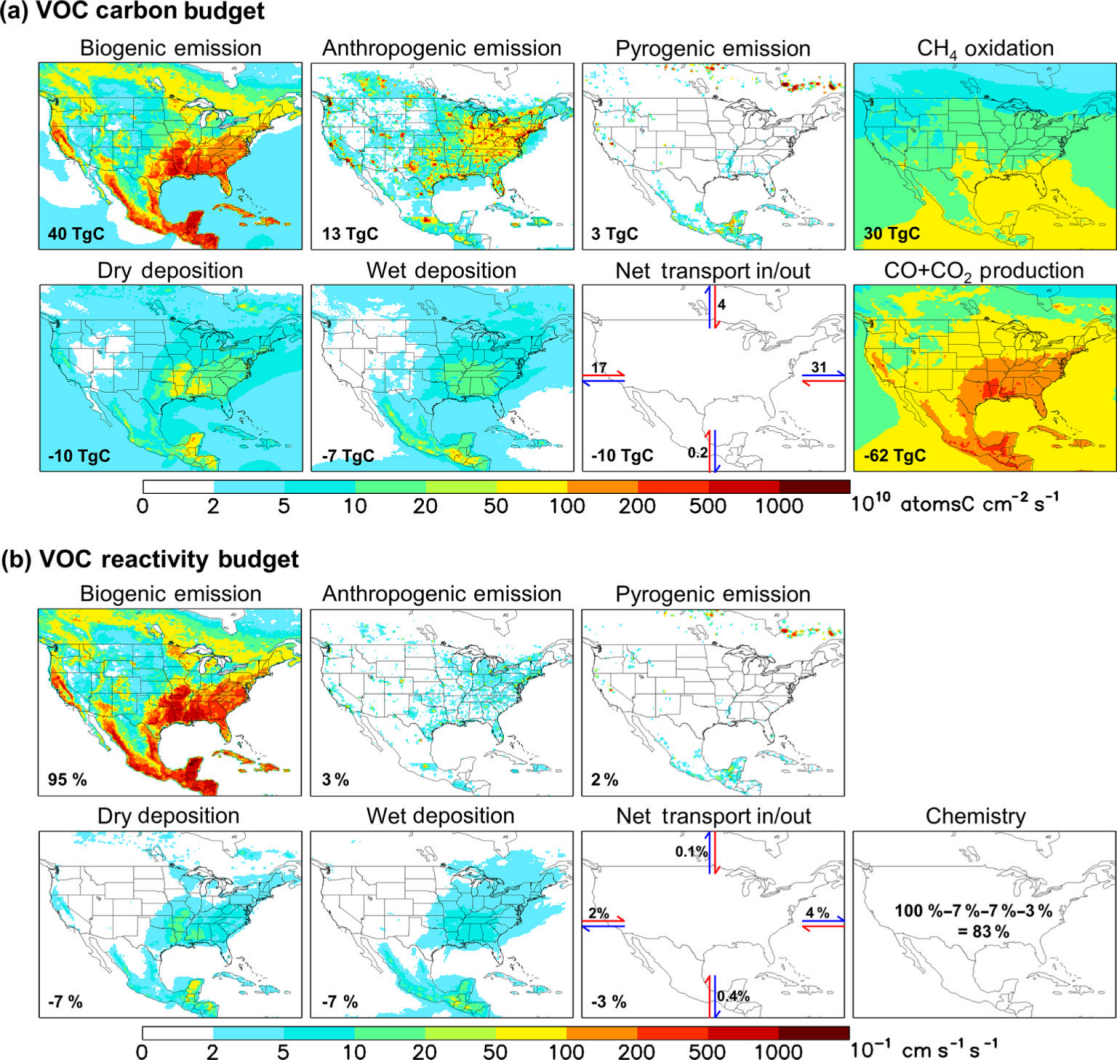
Annual VOC carbon (**a**) and reactivity (**b**) budgets over North America as simulated by GEOS-Chem for 2013. For panel (**a**) the annually integrated flux for each source or sink term is given inset. For panel (**b**) all VOC fluxes are weighted by the corresponding OH reaction rate coefficient at 298 K to derive a VOC reactivity budget. Values inset indicate the fraction of total emitted reactivity produced or removed by that source, sink, or transport process. Positive fluxes denote sources and negative fluxes denote sinks.

Figure 2a depicts the annual VOC budget (in C units) over North America in 2013 as simulated by GEOS-Chem. A buffer of 10 model grid boxes along each lateral boundary has been omitted to exclude unrealistic conditions near the edge of the nested domain. Total fluxes are indicated for each source and sink term, representing the sum over all grid boxes within the plotted region. The net transport flux in or out of the domain is estimated from the accumulated product of the daily average eastward or northward wind components and VOC number density at the boundaries. In this way, we achieve regional VOC-carbon closure to within 3 %.

We see in Fig. 2a that biogenic emissions are the dominant annual VOC-carbon source over North America, accounting for 71 % (40 Tg C) of the model total. Anthropogenic emissions account for 23 % (13 Tg C), while VOC emissions from fires can be important in particular locations and seasons but are minor when integrated over the domain as a whole (3 Tg C, 5 %). Prior studies have estimated that bio genic VOC emissions are 10–12x larger than anthropogenic emissions on a global basis (Safieddine et al., 2017; Gla-sius and Goldstein, 2016; Boucher et al., 2013; Guenther et al., 2012; Goldstein and Galbally, 2007); our results for North America, while indicating a greater relative importance for anthropogenic emissions than in the global mean, still show that biogenic VOC-carbon emissions are ~3x anthropogenic sources even in this industrialized region. Finally, while methane is not considered as a VOC for the purpose of our analysis, its oxidation generates formaldehyde and methyl hydroperoxide, corresponding to a VOC source of 30 Tg C yr^−1^ over our North American domain. Methane oxidation is thus > 2x larger as a nonmethane VOC source over this region than anthropogenic emissions, though this source is diffuse and not collocated with land-based fluxes.

**Figure 3.**
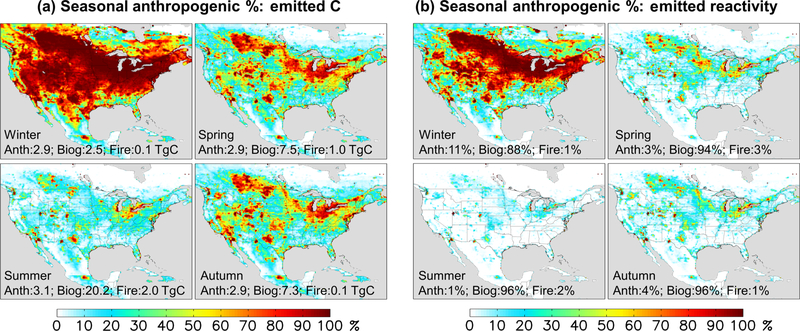
Seasonal anthropogenic contribution to total VOC-carbon emissions (**a**) and to total reactivity-weighted VOC emissions (**b**). Numbers inset indicate the domain-aggregated emissions (**a**) or domain-wide contribution to reactivity-weighted emissions (**b**) from anthropogenic, biogenic, and biomass burning sources.

During winter (Fig. 3a), we find in the model that anthropogenic sources account for the majority (54 %) of emitted VOC carbon over the domain as a whole; this fraction would be significantly higher if we were to exclude the US Gulf States, Mexico, and Central America, where substantial biogenic emissions persist throughout the year. However, during summer the modeled domain-wide anthropogenic contribution is only 12 %; then, it is only in the most polluted regions, where biogenic emissions are low, that anthropogenic emissions provide the main source of atmospheric reactive carbon.

Analogous sets of figures for NO_x_ are provided in the supplement (Figs. S5, S6).

### 4.2 Biogenic VOC emissions even more dominant on a reactivity basis

The predominance of biogenic over anthropogenic VOCs in North America is even more pronounced when we account for the chemical reactivity of the various species. A common metric for assessing this is the OH reactivity ( $\Sigma k_{i}n_{i}$ where *k_i_*, and *n_i_*, are the OH reaction rate coefficient and atmospheric number density for chemical *i*), which quantifies the OH loss rate associated with the ambient loadings of various species. In this paper, we use the term “VOC reactivity” to refer specifically to that portion of the OH reactivity driven by VOCs. A related, emissions-focused measure is the OH reactivity flux: i.e., $\sum k_{i}F_{\iota }$ , where *F_i_* is the surface flux for VOC *i* (in molecular units). Since the reactivity flux is equivalent to a (mixing-height scaled) time derivative of OH reactivity (Millet et al., 2018), it provides a direct measure of how a given surface flux affects ambient OH reactivity.

Figure 2b maps the modeled OH reactivity flux associated with biogenic, anthropogenic, and pyrogenic VOC emissions. We see that biogenic sources in the model account for 95 % of the annual reactivity-weighted VOC source over North America as a whole, with anthropogenic sources contributing just 3 %. This biogenic predominance continues throughout the year, with biogenic VOCs making up 88 % of the modeled domain-aggregated reactivity flux even during winter (though with strong spatial gradients; Fig. 3b). During summer, that fraction increases to 96 %.

There has been a substantial decrease in transportation-related VOC emissions over the past several decades in the US (McDonald et al., 2013; Parrish, 2006) (e.g., a factor of ~ 50–100 decrease was inferred over Los Angeles from 1960 to 2010; Warneke et al., 2012). According to current inventories (Fig. 3), anthropogenic emissions have declined to the point where biogenic emissions are the dominant summertime source of VOC reactivity even in many major North American cities. Only in a small number of pollution hotspots (Fig. 3) are anthropogenic emissions the main source of VOC-related OH reactivity driving summertime production of ozone and other secondary products.

### 4.3 Anthropogenic species comprise over half of the ambient VOC-carbon burden over most of North America

**Figure 4.**
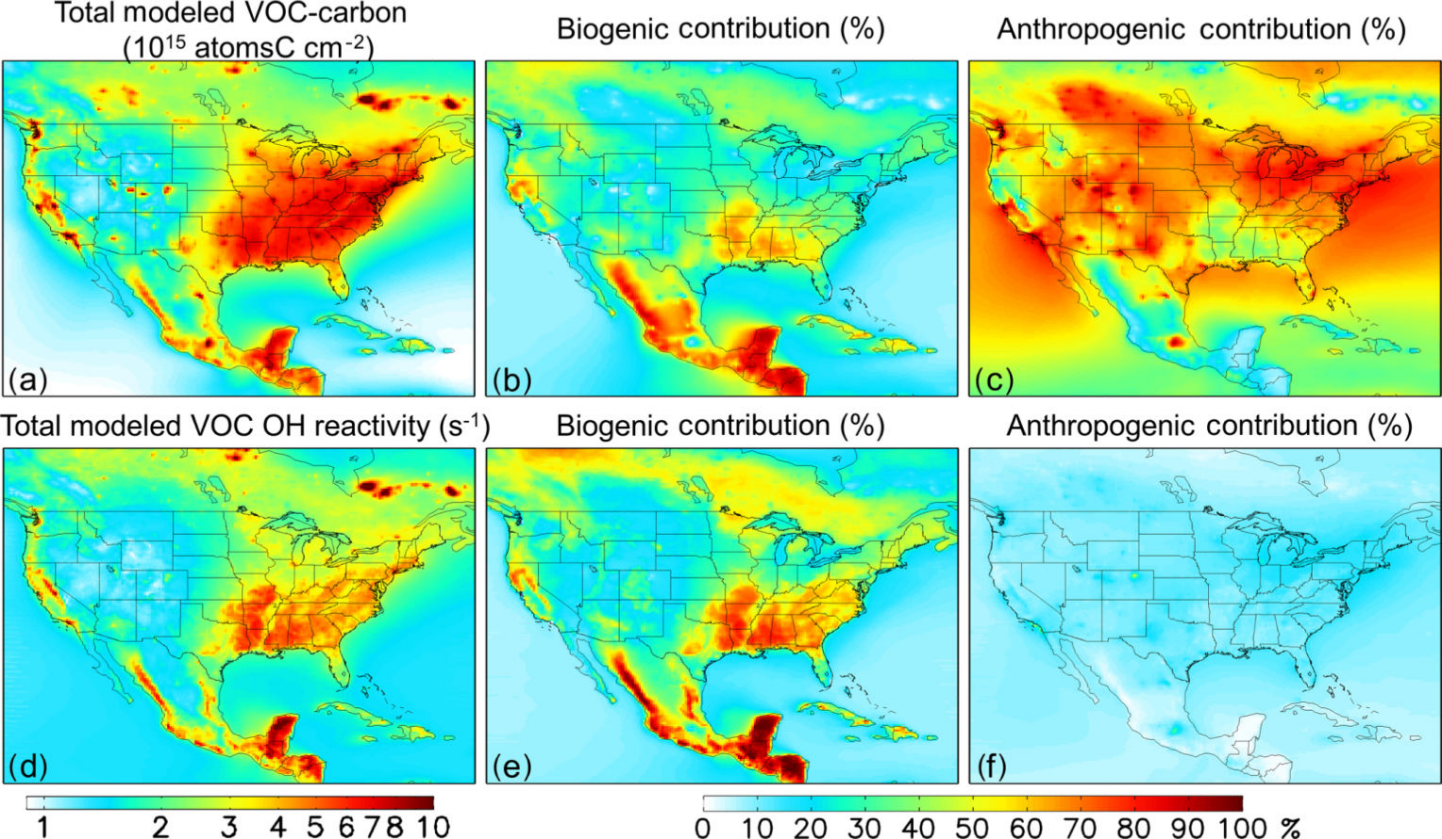
Distribution and source attribution of ambient VOC carbon and associated OH reactivity over North America. Panels (**a**) and (**d**): total VOC carbon and VOC-driven OH reactivity as simulated in the lowest model layer (below ~ 130 m). Panel (**b**) and (**e**): ambient VOC carbon and reactivity attributed to biogenic VOC emissions. Panel (**c**) and (**f**): ambient VOC carbon and reactivity attributed to anthropogenic VOC emissions. Source attributions are derived based on model sensitivity tests with 10 % modified anthropogenic or biogenic emissions, as described in the text.

Figure 4b, c, e, and f show the fractional contribution to the ambient near-surface VOC burden from anthropogenic and biogenic emissions. We quantify these contributions via model sensitivity tests with modified (−10 %) biogenic and anthropogenic VOC emissions; the contribution from each emission category is then obtained by dividing the relative change in ambient VOC carbon or reactivity by the relative emission perturbation. Partitioning the ambient VOC loading in this way provides an alternate framing of the VOC budget compared to the discussion above, which examined the VOC source flux magnitudes themselves.

While anthropogenic species make up only a small fraction of the total emitted VOC mass (~ 23 %; Fig. 2a), they account for more than half of the ambient near-surface VOC-carbon abundance over most of the North American domain (the median fraction in Fig. 4c is 57 %). This is due to the longer aggregate model lifetime for anthropogenic versus biogenic VOCs: because of this, away from major biogenic source regions the ambient VOC-carbon loading predominantly reflects anthropogenic species. However, many of these areas have relatively low total VOC-carbon loading (Fig. 4a). The corollary of the above finding is that the ambient VOC-driven OH reactivity is controlled by biogenic species, and this is also apparent in Fig. 4e and f.

### 4.4 Fate of reactive carbon over North America

The predominance of biogenic VOCs (in terms of total emitted VOC carbon) combined with their relatively short ensemble lifetime leads to a spatial correlation between biogenic VOC emissions and total VOC sinks (e.g., over the southeastern US; Fig. 2a). Figure 2a shows that of the 86 Tg C of nonmethane VOC added annually to the North American atmosphere through emissions, transport, and CH4 oxidation, 62 Tg C (72 %) is oxidized to CO+CO_2_ in the model. If we exclude the oxidation of methane (nearly 100 % of which goes on to form CO and CO2), then of the 56 Tg C yr^−1^ of primary VOCs emitted over North America, 32 Tg C yr^−1^ (57 %) is ultimately oxidized to CO+CO_2_ within the domain of Fig. 2. Oxidation of nonmethane VOCs therefore provides an atmospheric CO + CO_2_ source over this region greater than that from methane oxidation (30 Tg C yr^−1^), and greater than that from direct anthropogenic CO emissions (also 30 Tg C yr^−1^).

Other removal processes include deposition (dry, 10 Tg C yr^−1^; wet, 7 Tg C yr^−1^) and net transport out of the domain (10 Tg C yr^−1^). While global studies have found that wet deposition is a ~ 50 % larger sink of organic carbon than dry deposition (Safieddine et al., 2017; Kanakidou et al., 2012), the increased role for dry deposition found here is consistent with the higher continental coverage of our regional domain.

In the case of the VOC reactivity budget (Fig. 2b), we find in GEOS-Chem that chemical degradation is by far the largest sink (83 %) of emitted reactivity, with physical removal via deposition (14 %) and transport out of the domain (3 %) making up the remainder.

## 5 Observed versus predicted distribution of VOC carbon and reactivity over North America

In this section we use the aircraft campaigns described earlier to characterize the distribution of VOCs over North America and assess the ability of the GEOS-Chem model to capture that distribution in terms of total carbon loading and associated reactivity.

For each campaign we use the 1 min merge products provided by the NASA Langley Research Center (LaRC) and the NOAA Earth System Research Laboratory Chemical Science Division (ESRL CSD) (Table 1) and sample the model along the flight tracks at the time of measurement. Measurements have been filtered to remove fresh biomass burning (CH_3_CN > 0.2 ppbv) and pollution plumes (NO_2_ > 4 ppbv or NO_x_=NO_y_ > 0.4), and restricted to daytime measurements over continental North America. Model–measurement comparisons are performed for the PBL and FT based on unique grid-box–time-step combinations.

For the purposes of model–measurement comparison we restrict the observed VOCs to those that are explicitly simulated by GEOS-Chem (Millet et al., 2018). This restricted set of VOCs nonetheless encompasses those species believed to be most important in terms of abundance and reactivity (Heald et al., 2008), and allows an apples-to-apples compar ison between observations and model. For cases where multiple VOCs are measured together as a single quantity, the corresponding modeled VOCs are likewise summed. Similarly, measured VOCs are summed to match those that are lumped in the model.

VOC OH reactivities are calculated from the measured and simulated species concentrations and corresponding pressure- and temperature-dependent rate coefficients for reaction with OH. For species that are detected together but simulated separately, we use the modeled ratio to partition the measured sum in calculating the combined OH reactivity. For species that are lumped in the model but measured separately, we apply the bulk OH reaction rate coefficient from the model to the summed measurements.

In the case of C3 and C4 ketones and aldehydes, the model includes a dedicated tracer for acetone (ACET) and lumped tracers for ≥ C4 ketones (MEK) and ≥ C3 aldehydes (RCHO). On the other hand, these species are measured by PTR-MS as ∑(acetone + propanal) and ∑(MEK + butanal) and by TOGA as individual species. When analyzing the PTR-MS data we therefore partition the PTR-MS observations based on the median aldehyde V ketone ratio measured by TOGA during FRAPPÉ and DC3 (0.009 for propanal V acetone and 0.09 for butanal V MEK).

### 5.1 Total observed VOC carbon and reactivity over North America

**Figure 5.**
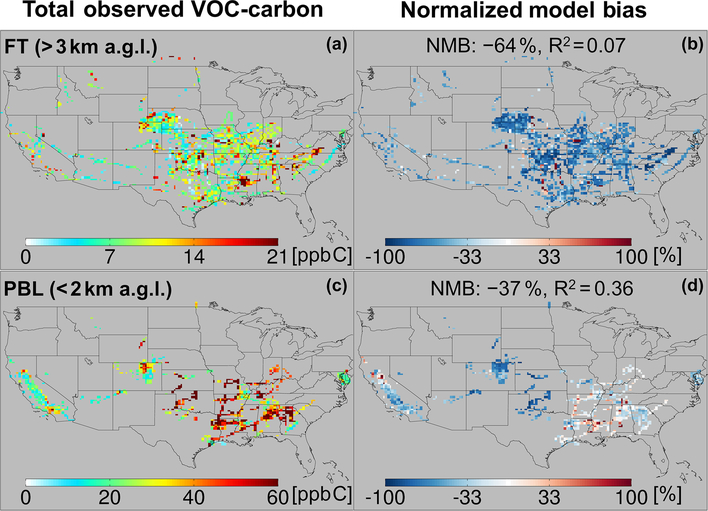
Total observed VOC-carbon loading (**a, c**) over North America in the free troposphere (> 3 km a.g.l.) and planetary boundary layer (< 2 km a.g.l.). In (**b, d**) the GEOS-Chem model simulation is compared to co-located aircraft observation with the normalized mean bias given inset. Note that the sampling season and instrument payload vary among campaigns.

Figure 5a and c show the resulting total VOC carbon as observed over North America, which averages 27 ppb C in the PBL when considering all the aircraft campaigns as a single statistical ensemble. However, the campaigns span a range of instrumental payloads, seasons, and locations: campaigns with the most comprehensive VOC instrument payloads and that occur during summer reveal total PBL VOC loadings generally > 60 ppb C, and up to 133 ppb C over the central and southeastern US. Campaigns over the northeastern and western US, with more limited VOC payloads, show PBL VOC loadings that average 20 ppb C and at times exceed 50 ppb C. Total VOC loadings in the FT (Fig. 5) drop by a factor of ~3 or more from those in the PBL across all environments, with an ensemble spatial mean of 9 ppb C.

The observed VOC-carbon loadings summarized above and plotted in Fig. 5 are broadly similar to those reported over the US by Heald et al. (2008) (averaging 8–84 ppb C with 83 %–97 % in the gas-phase at 273 K and 1013 hPa), who synthesized the gas- and aerosol-phase organic carbon observations up to that time. However, observations over the US used in that study were primarily from ground-based campaigns. The 10 airborne studies carried out since then and used here allow a more comprehensive spatial description of VOCs across the North American airshed. The combined dataset employed here also includes a number of ad ditional multifunctional VOCs that can now be quantified thanks to measurement advances in the intervening decade (Glasius and Goldstein, 2016).

**Figure 6.**
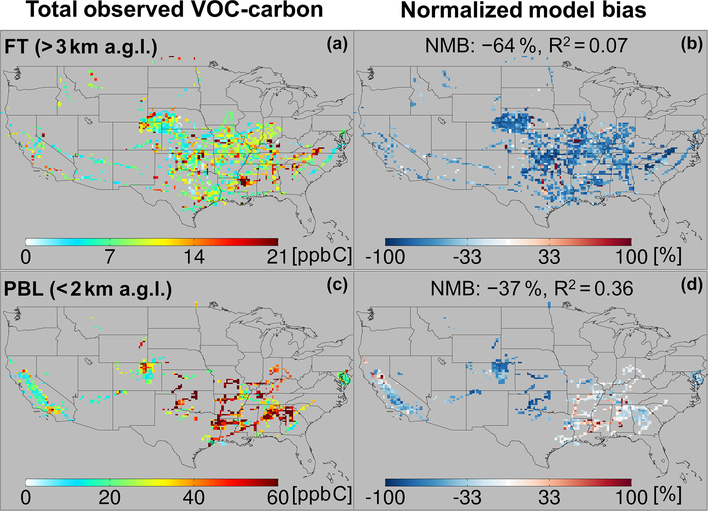
Total observed VOC reactivity (**a, c**) over North America in the free troposphere (> 3 km a.g.l.) and planetary boundary layer (< 2 km a.g.l.). In (**b, d**), the GEOS-Chem model simulation is compared to co-located aircraft observation with the normalized mean bias given inset. Note that the sampling season and instrument payload vary among campaigns.

Figure 6a and c show the total OH reactivity arising from the set of observed VOC. The aggregated spatial mean VOC reactivity is 2 s^−1^ in the PBL, declining to 0.13 s^−1^ in the FT. Compared to the VOC-carbon loading, the reactivity has a much larger vertical falloff (10–20 times decrease from the PBL to the FT), and greater spatial variability within the PBL. The observed VOC reactivity within the PBL is generally > 6 s^−1^ over the southeastern US, 2–6 s^−1^ over the northeastern US, and < 2 s^−1^ over the central and western US. The highest observed VOC reactivity (24 ^-?^1) over the southeastern US is comparable to ground-based measurements in that region (10–25 ^-?^1) during the SOAS study (Feiner et al., 2016; Kaiser et al., 2016).

The importance of biogenic VOCs for reactive carbon loading and, especially, reactivity in the PBL is evident in the maps shown in Figs. 5–6. For example, Fig. 6 shows sharply defined areas of elevated VOC reactivity in the PBL over the forests of the southeastern US, with strong horizontal gradients and much lower observed reactivity elsewhere. Similar patterns, though less starkly defined, are evident in the measured VOC-carbon distribution (Fig. 5). The highly reactive nature of many biogenic VOCs (especially isoprene and some of its oxidation products) explain their disproportionate impact on reactivity given their relative abundance, as well as the much larger spatial gradients for VOC reactivity than for total VOC carbon.

### 5.2 Speciated drivers of ambient VOC carbon and reactivity

**Figure 7.**
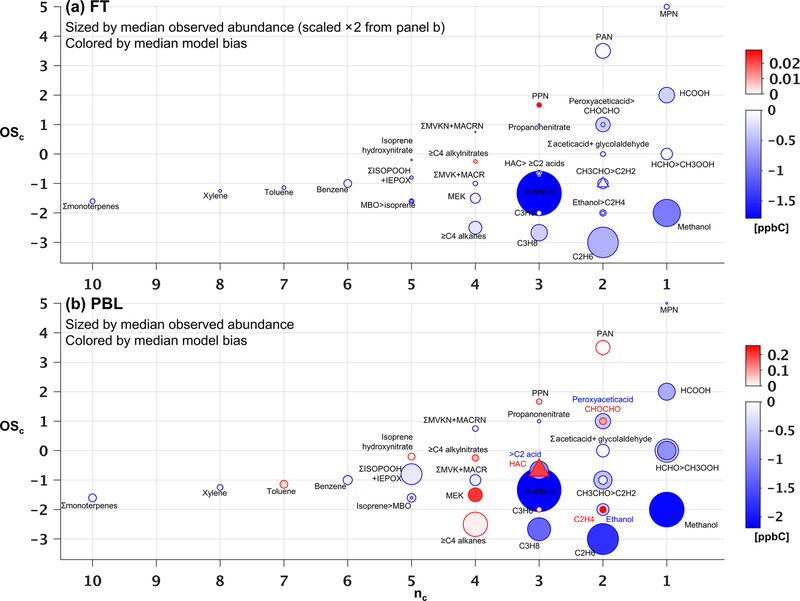
Observed versus predicted VOC carbon as a function of carbon oxidation state (OS_c_) and number of carbon atoms (*n*_c_). Each circle indicates a single VOC (or lumped category for those that are measured or modeled collectively). Symbols are sized according to the observed median abundance (ppbC) of each species in the FT (**a**) and in the PBL (**b**, note altered size scaling from **a**). Triangles are used when co-located circles are too close in size to distinguish, and symbols are colored according to the median absolute model bias in each case. For overlapping species, the more abundant of the two is indicated with “>”.

**Figure 8.**
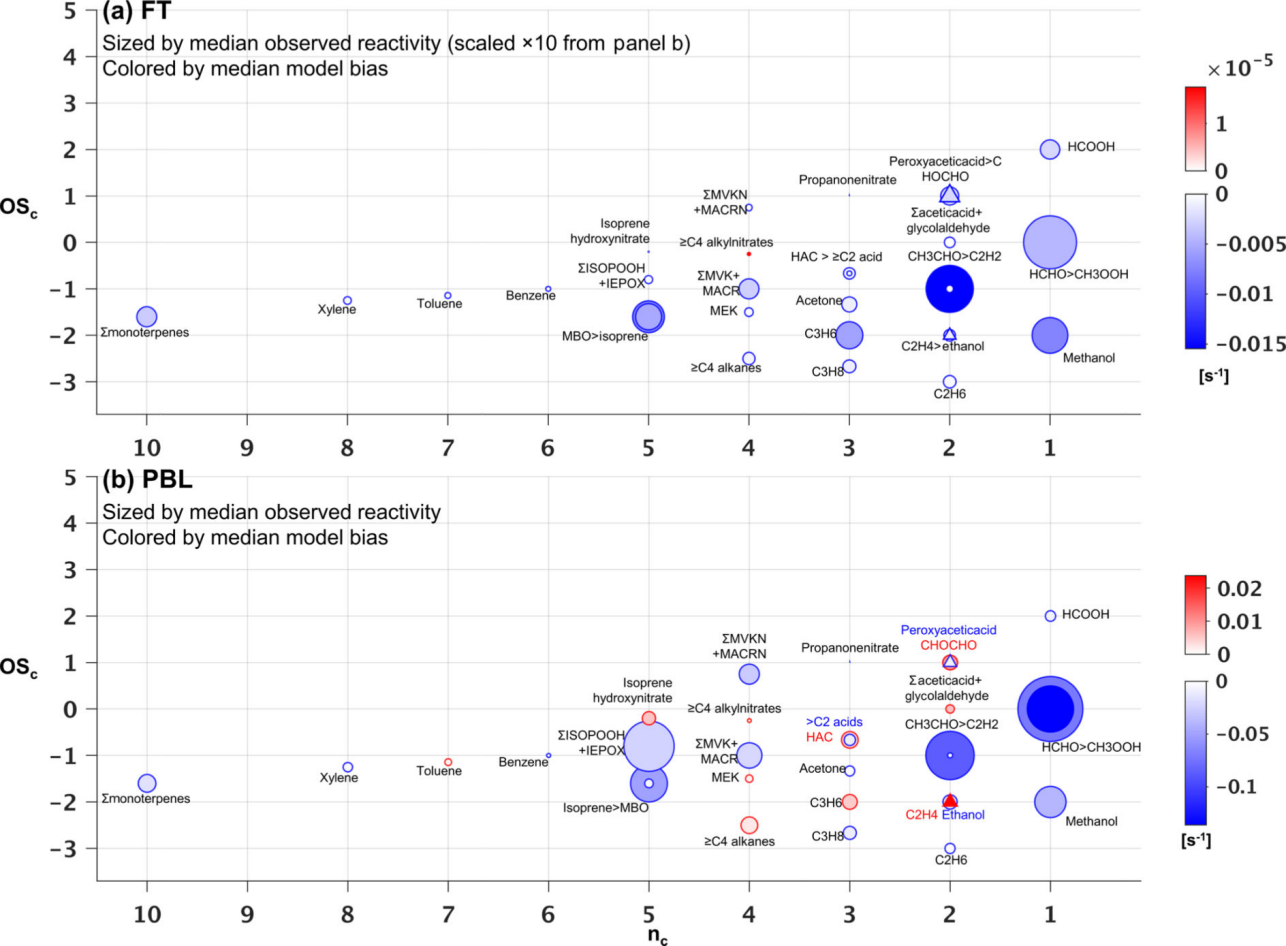
Observed versus predicted VOC reactivity as a function of carbon oxidation state (OS_c_) and number of carbon atoms (*n*_c_). Each circle indicates a single VOC (or lumped category for those that are measured or modeled collectively). Symbols are sized according to the observed median reactivity (s^−1^) of each species in the FT (**a**) and in the PBL (**b**, note altered size scaling from **a**). Triangles are used when co-located circles are too close in size to distinguish, and symbols are colored according to the median absolute model bias in each case. For overlapping species, the more abundant of the two is indicated with “>”.

Figures 7 and 8 show the species driving ambient VOC carbon and reactivity as a function of their carbon oxidation state (OSc) and size (carbon number, *n*_c_) (Kroll et al., 2011). Within the PBL (Fig. 7b), we find that the total mean VOC carbon is largely driven by small and relatively reduced VOCs (e.g., acetone, methanol and alkanes), though some more oxidized species (e.g., formic acid, methyl hydroperoxide, formaldehyde, other isoprene oxidation products) also make significant contributions. These smaller VOCs would represent an even larger portion of the total molar VOC-loading.

In the FT (Fig. 7a), mean abundances decline by approximately a factor of 2 or more for all measured VOCs relative to the PBL. Here, a few small, reduced (low-OS_c_), and relatively long-lived species dominate the overall VOC-carbon loading, with acetone, methanol, and ethane (τ ~ 12–50 days at OH = 10^6^ molecule cm^−3^) together averaging 6.4 ppb C, compared to only 3.6 ppb C for the mean sum of all other observed species.

However, ambient OH reactivity is driven by a different set of VOCs. Figure 8 shows that within the PBL, formaldehyde (0.34 s^−1^), acetaldehyde (0.19 s^−1^), isoprene hydroxy hydroperoxides + epoxides (0.21 s^−1^), methyl hydroperox ide (0.17 s^−1^), and isoprene (0.11 s^−1^) make the largest contributions to the mean observed VOC reactivity. Compared to the case for VOC-carbon loading (Fig. 7b), we see in the reactivity distribution a more prominent role for a number of higher-nc (and more reactive) compounds.

On average, the observed VOC reactivity is more than a factor of 10 lower in the FT than in the PBL, with formaldehyde (0.03 s^−1^) and acetaldehyde (0.02 s^−1^) still making the largest contributions to the total. Whereas the FT VOC-carbon loading is dominated by a few small VOCs (Fig. 7a), Fig. 8a shows that the FT VOC reactivity is provided by a wider suite of species due to the offsetting effects of abundance and lifetime. In other words, we see important FT reactivity contributions (in the mean) from both highly reactive (but low-abundance) VOCs such as isoprene, and from less-reactive (but highly abundant) VOCs such as methanol.

### 5.3 Accuracy of CTM-predicted VOC carbon and reactivity

Figures 5 and 6 also portray the ability of the GEOS-Chem CTM to represent the measured distribution of VOCs over North America. In the PBL, the model exhibits significant skill at capturing atmospheric variability in VOC carbon and reactivity: spatial model–measurement *R*^2^ values are 0.36 and 0.54, respectively. The same is not true in the FT, where the model–measurement correlations are *R*^2^ < 0:1 for both VOC carbon and VOC OH reactivity. This lack of explanatory power suggests that the primary drivers of VOC abundance and reactivity in the FT are not well-understood or well-represented in current models.

We also see in Figs. 5 and 6 that the model tends to underestimate the observed VOC carbon and reactivity in the PBL across most of the sampled environments, with a normalized mean bias (NMB) of −37 % and −34 %, respectively. This corresponds to a mean reactive carbon underestimate in the PBL of 10 ppb C and a reactivity underestimate of 0.6 s^−1^. A bias of this magnitude is equivalent to ~2x the reactivity of methane (at 2 ppm) or 0:5x that of CO (at 200 ppb) and is therefore important for accurately representing atmospheric OH chemistry and ozone production.

While on average the CTM underpredicts the abundance and reactivity of VOCs in the PBL, this is not the case everywhere. There are areas shown in Figs. 5 and 6 where the model either agrees with the observations or is too high – in particular over the northern Sacramento Valley and the southeastern US. Regarding the former, large methanol and acetaldehyde emissions from rice fields, with strong enhancements after flooding, were previously inferred based on the same CalNex observations over the Central Valley (Peischl et al., 2012; Warneke et al., 2011). Indeed, we find here a model overestimate of total VOC carbon for this region before flooding and a low bias after flooding, suggesting that agricultural VOC emissions are not currently well-represented in the model. On the other hand, over the southeastern US, where biogenic emissions predominate and VOC loading is highest across all sampled areas, both the PBL VOC carbon (observed mean of 48 ppb C) and VOC reactivity (4.5 s^−1^) are captured by the model with low mean bias (< 14 % for both).

In contrast to the PBL where both positive and negative model discrepancies occur, aloft in the FT the model exhibits a large negative bias for both VOC carbon (−64 %) and reactivity (−63 %) that manifests essentially everywhere. Such a severe discrepancy has implications for our understanding of FT HOx cycling (Brune et al., 2018; Mao et al., 2009), ozone production at higher altitudes where its climatic effects are strongest (Apel et al., 2015; Bertram et al., 2007), and possibly secondary organic aerosol loading (Bianchi et al., 2016; Cappa, 2016; Kirkby et al., 2016; Trostl et al., 2016; Heald et al., 2005). We explore potential causes for these observed discrepancies in Sect. 6.

Given the range in measurement years spanned by the aircraft measurements, we performed a set of 1-month simulations spanning multiple years to assess the potential impact of interannual variability on these findings. Results (see Fig. S7 and text following) suggest that the key features of the model–measurement comparisons discussed here are robust across years.

### 5.4 Key VOCs driving model biases in atmospheric VOC carbon and reactivity

Figure 7b shows that the overall low model bias for VOC carbon in the PBL manifests for 23 out of 34 individual VOCs, with these exhibiting normalized biases ranging from −1 % to −90 % (Figs. S8b and S9b). In general, the largest absolute carbon biases are seen for the more abundant VOCs (Fig. 7b), and the largest reactivity biases for the more reactive VOCs (Fig. 8b). Just two compounds (acetone and methanol) account for almost half of the mean negative VOC-carbon bias seen in the PBL (4.3 of 9 ppb C). For VOC reactivity, four compounds (methyl hydroperoxide, acetaldehyde, formaldehyde, and isoprene) together account for 70 % of the mean model bias in the PBL (−0:34 of −0:47 s^−1^).

Aloft in the FT (Figs. 7a and 8a), we see appreciable relative biases manifest across nearly all model compounds (ranging from −7 % to −100 %; Figs. S8a and S9a), with 29 out of 34 VOCs biased low in the model by more than a factor of 2. Acetone, methanol, and ethane are predominant in driving the overall model VOC-carbon underestimate: these three species have a combined model bias of −3:3 ppb C, versus a total of only −2:1 ppb C for all other underestimated VOCs combined. Significant discrepancies in model-simulated FT VOC reactivity are driven by both abundant but less reactive VOCs, and by reactive (but less abundant) VOCs, with acetaldehyde having by far the largest absolute bias overall (−0:015 s^−1^).

The above comparisons point to research priorities for improving current model representations of atmospheric VOCs. Along with highly abundant VOCs (such as acetone, methanol, and ethane), acetaldehyde, formaldehyde, isoprene (plus its oxidation products), and methyl hydroperoxide drive a large fraction of total VOC reactivity and associated model biases. Advancing our current ability to model the sources, chemistry, and physical removal of this relatively small number of species could substantially improve predictions of VOC carbon and reactivity distributions.

## 6 Role of vertical transport in driving a persistent model VOC underestimate in the free troposphere over North America

**Figure 9.**
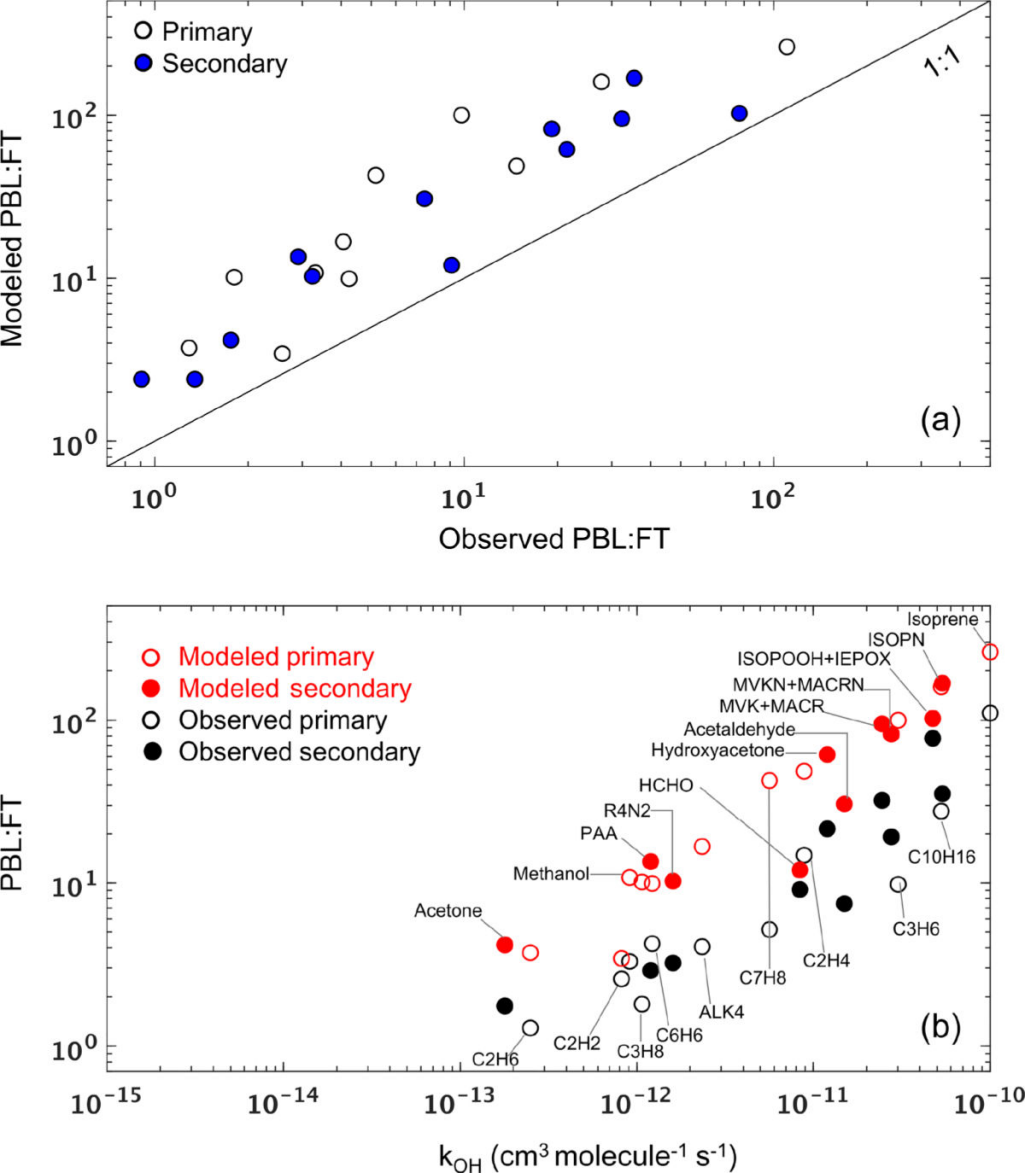
(**a**) Modeled versus observed mean PBL: FT ratio (mixing ratio units) for each VOC during the SEAC^4^RS campaign. Each data point represents a single VOC, and the 1: 1 line is also shown. (**b**) Modeled and observed mean PBL: FT ratio for VOCs during SEAC^4^RS as a function of their OH reaction rate coefficient at 298 K. In both panels, unfilled and filled symbols indicate species with predominantly primary and secondary sources, respectively.

In Sect. 5 we demonstrated that VOC abundance and reactivity are consistently underestimated by the model in the free troposphere across environments and compounds. Potential explanations for these missing FT VOCs include chemical effects (e.g., model biases in FT VOC production and loss rates) as well as dynamical effects (e.g., model biases in PBL-FT mixing). To help distinguish between these two, we plot in Fig. 9a the modeled versus observed mean PBL: FT ratio (mixing ratio units) for each VOC across the entire SEAC^4^RS campaign. We see that all data fall above the 1: 1 ratio line, showing that the model is overestimating the PBL: FT ratio to a similar degree across all VOCs regardless of source, lifetime, and chemical properties. This consistency across compounds points to a misdiagnosis of PBL ventilation as a likely explanation for the persistent VOC underestimate in the FT (at least over the SEAC^4^RS domain), since other tenable mechanisms would not be expected to affect all VOCs in such a consistent way. In particular, (i) a missing FT photochemical VOC source would not explain the PBL: FT discrepancy seen for primary VOCs, (ii) a model bias in dry deposition or wet scavenging would differentially affect polar and soluble versus nonpolar and less soluble species, and (iii) a model OH bias would impact reactive and longer-lived species to differing degrees. Findings similar to those shown in Fig. 9a are obtained for other campaigns over the southern and eastern US (SENEX, DISCOVER-AQ DC, DISCOVER-AQ TX) but not consistently elsewhere (DC3, DISCOVER-AQ CO, FRAPPÉ, DISCOVER-AQ CA, CalNex). Since the southeastern US is the major source of North American VOC carbon and reactivity (Fig. 2), such a mixing bias would yield a significant model underestimate of the total amount of reactive organic carbon that is transported to the North American FT.

We can explore this issue further by considering a two-box model to conceptualize VOC partitioning between the PBL and FT. In that case, for an example VOC that is directly emitted and then subject to chemical loss by OH, PBL-FT mixing, and deposition (PBL only), the steady-state PBL: FT ratio would be linearly related to the OH rate coefficient kOH with a slope determined by OH and by the PBL ventilation rate, and with an intercept determined by the PBL-FT mixing rates. Figure S10 shows that the same holds for secondary VOCs. While dilution with PBL and FT background air will also affect the PBL: FT ratio, its effect in this simplified framework will diminish as the extent of the domain considered increases, and for shorter-lived species.

Of the aircraft campaigns considered, SEAC^4^RS comes closest to the above approximation due to the larger spatial domain sampled by the DC8 aircraft. The modeled and observed PBL: FT ratios for this campaign are plotted in Fig. 9b as a function of kOH. For both model and measurements, there is an approximately linear relationship, with the model generally capturing the observed PBL: FT vs. kOHslope. However, with only a couple of exceptions (e.g., HCHO, C_2_H_2_), there is a clear offset between the two populations that manifests in a consistent way for both primary and secondary VOCs and across lifetimes. The offset persists even after correcting for a potential 40 % PBL depth overestimate (Zhu et al., 2016) in the GEOS fields (Fig. S11). The same conclusions are obtained if we instead examine the PBL: (PBLC FT) or (PBLC FT): PBL ratios to minimize any potential influence from spurious ratios caused by near-detection-limit VOC measurements (not shown). Overall, the above comparisons implicates PBL: FT mixing as a likely player in the pervasive model VOC biases found in the FT.

These findings are consistent with those of Yu et al. (2018), who diagnosed inadequate vertical transport in the current off-line configuration of the GEOS-Chem CTM. Yu et al. (2018) identified as causes (i) the off-line convective transport scheme (leading to a +10 % bias in modeled ^222^Rn at the surface, and a −5 % bias in the upper troposphere), and (ii) off-line archiving of the meteorological fields (+5 % model surface bias and −20 % upper troposphere bias). Fixing these issues would therefore reduce the errors found here for VOCs in the free troposphere (~ 60 % mean low bias) but worsen the aggregated model performance in the PBL (~ 30 % mean low bias). In that case, we would likely see in the model a more consistent low VOC bias throughout the troposphere, which would then indicate errors in overall VOC emissions or other processes.

## 7 Role of biogenic versus anthropogenic sources in driving model biases for key oxygenated VOCs in the North American boundary layer

Section 5 demonstrated the critical role that certain light OVOCs (e.g., formaldehyde, acetaldehyde, methanol, acetone, methyl hydroperoxide) play in defining atmospheric VOC-carbon loading and associated reactivity, and in driving model biases in those quantities. We see in Fig. 7 that while the GEOS-Chem model underestimates the abundance of most OVOCs in the PBL, some species are overestimated (analogous discrepancies are seen in the average vertical profiles; Figs. S12–S21). We therefore investigate in this section the likely role of biogenic versus anthropogenic sources in driving the observed model biases for key OVOCs.

To this end, a unique pair of biogenic (BOVOC) and anthropogenic (AOVOC) source tracers was developed for each OVOC based on the mixing ratio difference along the flight track for that species between the model base case and simulations with either (i) all biogenic VOC emissions perturbed by 10 %, or (ii) all anthropogenic VOC emissions perturbed by 10 % (see Sect. 4.3). BOVOC thus represents the integrated influence of direct biogenic emissions plus oxidation of biogenic precursors for a given OVOC along the aircraft flight track, based on the model simulation. AOVOC is likewise a marker for the combined influence of primary plus secondary anthropogenic sources. We find that the above tracers are best able to capture the observed in-PBL OVOC variance for the SEAC^4^RS, SENEX, and DISCOVER-AQ TX campaigns (Table S6), arguing that the allocation of model VOC sources has the highest spatial reliability over the southeastern US region. We therefore focus our source-tracer interpretation on these specific campaigns.

**Figure 10.**
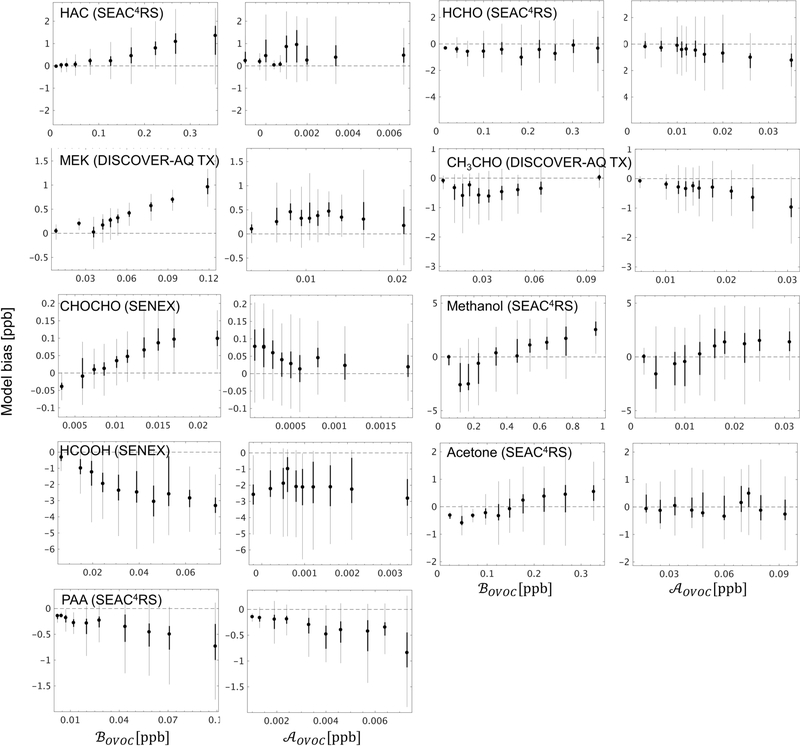
GEOS-Chem model bias for select OVOCs in the boundary layer (< 1 km here), binned according to the contribution from biogenic (BOVOC) and anthropogenic (AOVOC) sources to the overall abundance. BOVOC and AOVOC represent the integrated influence of primary + secondary biogenic and anthropogenic sources (respectively) for a given OVOC along the aircraft flight track based on the model simulation, as described in the text. The 10 plotted bins each represent an equal number of data points for a given OVOC, with the box plots indicating the corresponding median (filled circle), interquartile range (thick line), and 99 % confidence interval (thin line).

Figure 10 plots the model bias for select OVOCs as a function of BOVOC and AOVOC and shows that in several cases the model OVOC errors exhibit a clear relationship with one (or both) of these source tracers. For example, the positive model bias seen previously (Fig. 7) for hydroxyacetone (HAC), methyl ethyl ketone (MEK), and glyoxal (CHO-CHO) is strongly correlated with the biogenic source tracer BOVOC for each species, with the largest model overestimates occurring when BOVOC is high. This points to a current model overestimate of the biogenic sources of HAC, MEK, and CHOCHO, either due to biases in their precursor emissions (e.g., Kaiser et al., 2018; Zhu et al., 2016; Wolfe et al., 2015) or in their chemical formation mechanisms (e.g., Chan Miller et al., 2017; Li et al., 2016). Model sink errors may also play a role (e.g., Curry et al., 2018); however, to explain the results in Fig. 10, such biases would need to be spatially correlated with emissions.

Conversely, in the case of formic acid (HCOOH) the model bias becomes more negative with increasing biogenic influence (consistent results are obtained with either the UW or NOAA measurements, Fig. S22), which is consistent with earlier findings (Alwe et al., 2019; Millet et al., 2015; Stavrakou et al., 2012) pointing to an underestimated biogenic source of HCOOH or its precursors over the southeastern US. The negative model bias seen for PAA (Fig. 7) increases with both BOVOC and AOVOC (Fig. 10), which may indicate a generic underestimate of PAA production across biogenic and anthropogenic VOCs, or an overestimation of its chemical loss.

Findings for other OVOCs tend to be less clear and/or less consistent across these campaigns. Acetaldehyde (CH3CHO) is biased low in the model, on average, across the aircraft campaigns (Fig. 7), and there is some indication that this is partly due to underrepresented anthropogenic sources (Figs. 10, S22–S24). Acetone and methanol are strongly underestimated by the model (Fig. 7), which drives a significant part of the overall model VOC-carbon bias over North America. However, Fig. 10 shows that while the model bias is negative under low values of BOVOC, it is positive under high values of BOVOC (this is specifically the case for SEAC^4^RS and DISCOVER-AQ TX; Figs. S22–S24): this may indicate a model overestimate of direct biogenic emissions combined with an underestimate of regional background concentrations or of other sources.

## 8 Summary

We performed an integrated analysis of the atmospheric VOC budget over North America based on an ensemble of recent airborne observations interpreted with an updated version of the GEOS-Chem CTM. A total of 86 Tg C of nonmethane VOCs is added annually to the North American atmosphere in the model through emissions (biogenic: 40 Tg C; anthropogenic: 13 Tg C; fires: 3 Tg C) and CH_4_ oxidation (30 Tg C yr^−1^). Of that, 62 Tg C is oxidized to CO + CO2, with the rest removed by deposition (dry: 7 Tg C yr^−1^; wet: 10 Tg C yr^−1^) and net transport out of the domain (10 Tg C yr^−1^).

The simulated North American VOC budget shows the dominance of biogenic VOC emissions on a carbon basis (71 %) and even more markedly on a reactivity basis (95 %). Anthropogenic emissions provide the dominant summertime source of VOC carbon and reactivity only in a fairly small number of pollution hotspots, and annually these emissions are > 2x smaller as a source of nonmethane VOC over North America than is methane oxidation. Nevertheless, anthropogenic VOCs provide more than half of the ambient VOC-carbon burden over the majority of the region due to their longer average lifetime relative to biogenic species.

While on-road VOC emissions in North America have undergone a substantial decrease in the past few decades (McDonald et al., 2013; Warneke et al., 2012), recent studies have pointed to the importance of (i) emerging VOC sources from oil and gas facilities (Li et al., 2017; Pfister et al., 2017), (ii) volatile chemical products (McDonald et al., 2018), and (iii) unexpectedly large urban OVOC fluxes (Karl et al., 2018). It is possible that such sources are not well captured in current inventories such as those used here, which in turn could alter the budget understanding above. These areas require further research to better understand the importance of such emissions for atmospheric chemistry, and to test and improve their representation in models.

Based on the collective aircraft observations, we find that total ambient VOC carbon over North America is dominated by small and relatively reduced VOCs (e.g., acetone, methanol, alkanes), along with some oxidized species (e.g., formic acid, methyl hydroperoxide, formaldehyde, other isoprene oxidation products) that are also substantial VOC-carbon reservoirs in the planetary boundary layer (PBL). In the free troposphere (FT), acetone, methanol, and ethane together average 6 ppb C over the ensemble of airborne data, compared to only 4 ppb C for the sum of all other measured VOCs. Formaldehyde and acetaldehyde provide the largest source of VOC reactivity, on average, in both the PBL and FT, with a range of other reactive (but less abundant) and abundant (but less reactive) species also making significant contributions.

The GEOS-Chem CTM with state-of-science VOC treatment captures a significant portion of the observed ambient variability for VOC carbon (*R*^2^ D 0:36) and reactivity (0.54) in the PBL, but not in the FT (0.07 and 0.04) – suggesting that the main factors influencing VOC abundances in the FT are inadequately represented in current models. The GEOS-Chem model exhibits both underestimates and overestimates of the observed VOC carbon and reactivity in the PBL, depending on location, with an overall normalized mean bias of −37 % (carbon) and −34 % (reactivity). This mean bias is equivalent to ~ 2x the reactivity of methane at 2 ppm or 0.5x that of CO at 200 ppb and is therefore important from the point of view of accurately predicting OH chemistry and ozone production.

In the FT, the model exhibits a persistent low bias (~ 60 %) for VOC carbon and reactivity that manifests essentially everywhere. A comparison of modeled versus observed PBL: FT VOC concentration ratios over the southeastern US suggests that inadequate PBL ventilation in the model may play a role in driving the observed FT biases. Recent work has sought to improve CTM transport performance through improved spatial resolution (e.g., Zhuang et al., 2018; Yu et al., 2016), through use of a cubed-sphere rather than regular Cartesian grid (e.g., Eastham et al., 2018; Yu et al., 2018), and by integration into Earth system models with online coupled meteorology (e.g., Hu et al., 2018; Long et al., 2015). Further work is needed to specifically assess model treatment of PBL-FT coupling (e.g., using PAN: NOx or other diagnostic quantities) and PBL depths to improve tracer simulations in the FT.

We used a source tracer analysis to investigate the likely role of biogenic versus anthropogenic sources in driving model biases for key oxygenated VOCs. Results point to a current overestimate of the (primary + secondary) biogenic sources of hydroxyacetone, methyl ethyl ketone, and glyoxal and an underestimate of the biogenic sources of formic acid. Results also suggest a possible underestimate of the anthropogenic sources of acetaldehyde, along with an underestimate of peroxyacetic acid production across both biogenic and anthropogenic precursors. Finally, we find that a relatively modest number of individual VOCs (acetone, methanol, ethane, acetaldehyde, formaldehyde, isoprene + oxidation products, methyl hydroperoxide) drive a significant fraction of the total ambient VOC carbon and reactivity (and associated model biases) across many environments. These species therefore merit further research to better understand their budgets and to improve model representation of VOC chemistry and the resulting effects on SOA, O_3_, and other oxidants.

## Supplementary Material

SI

## Figures and Tables

**Table 1.** Overview of aircraft campaigns used here*.

	Aircraft platform	Aircraft ceiling	Timeframe	Sampling region	Campaign overview and data DOI if applicable
CalNex	NOAA WP-3D	7600 m	May–July 2010	California and offshore	Ryerson et al. (2013)
DC3	NASA DC-8 NSF/NCAR GV	12 500m 15 500m	May–June 2012	Northeastern Colorado, west Texas to central Oklahoma, and northern Alabama	Barth et al. (2015), DC3 Science Team (2013)
SENEX	NOAA WP-3D	7600m	June–July 2013	Southeastern US	Warneke et al. (2016)
SEAC^4^RS	NASA DC-8	12 500m	August–September 2013	Southeastern US and Gulf of Mexico	Toon et al. (2016), SEAC^4^RS Science Team (2013)
DISCOVER-AQ	NASA P-3B	8500 m	June–July 2011 January–February 2013 September 2013 July–August 2014	Baltimore–Washington, D.C., San Joaquin Valley, California, Houston, Texas, and Denver, Colorado	Crawford and Pickering (2014), DISCOVER-AQ Science Team (2014)
FRAPPÉ	NCARC-130	7900 m	July–August 2014	Northern Colorado	Pfister et al. (2017)

* See measurement details in Table S1 (O’Sullivan et al., 2018; Treadaway et al., 2018; Lerner et al., 2017; Min et al., 2016; M. Müller et al., 2014, 2016; Cazorla et al., 2015; Richter et al., 2015; Lee et al., 2014; Yacovitch et al., 2014; Kaser et al., 2013; DiGangi et al., 2011; Fried et al., 2011; Zheng et al., 2011; Apel et al., 2010; Pollack et al., 2010; St Clair et al., 2010; Weibring et al., 2010; Wooldridge et al., 2010; Gilman et al., 2009; Hottle et al., 2009; Osthoff et al., 2008; de Gouw and Warneke, 2007; Huey, 2007; Kim et al., 2007; Crounse et al., 2006; Slusher et al., 2004; Blake et al., 2003; Schauffler et al., 2003; Wisthaler et al., 2002; Colman et al., 2001; Ryerson et al., 1999, 1998; Weinheimer et al., 1994).
