# Supplementary material for: On the sources and sinks of atmospheric VOCs: an integrated analysis of recent aircraft campaigns over North America
Source: Atmos Chem Phys. Author manuscript; Available in PMC 2021 Mar 8. (PMC7939023; doi:10.5194/acp-19-9097-2019)
Supplement: SI [file NIHMS1669556-supplement-SI.pdf]

Supplement of Atmos. Chem. Phys., 19, 9097–9123, 2019
https://doi.org/10.5194/acp-19-9097-2019-supplement
© Author(s) 2019. This work is distributed under
the Creative Commons Attribution 4.0 License.

Supplement of

On the sources and sinks of atmospheric VOCs: an integrated analysis of recent aircraft campaigns over North America

Xin Chen et al.

Correspondence to: Dylan B. Millet (dbm@umn.edu)

The copyright of individual parts of the supplement might differ from the CC BY 4.0 License.

Table S1. Gas-phase species and instrumentation used here.

Campaign	Species involved in this work	Measurement technique	Measurement reference
CalNex	isoprene, monoterpenes, benzene, toluene, C8 aromatics<br>methanol, acetone, methyl ethyl ketone, formaldehyde, acetaldehyde, MVK+MACR<br>acetonitrile	PTR-MS	de Gouw and Warneke (2007)
	2-$BuONO_2$, 3-$PenONO_2$, 2-$PenONO_2$, 3-methyl-2-$BuONO_2$<br>C_2H_6, C_3H_8, i-butane, n-butane, i-pentane, n-pentane, n-hexane, n-heptane, n-octane, 2,3-dimethylbutane, 2-methylpentane, 3-methylpentane, C_2H_4, propene, 1-butene, trans-2-butene, cis-2-butene, C_2H_2	WAS	Colman et al. (2001), Schauffler et al. (2003)
	PAN	CIMS	Zheng et al. (2011), Osthoff et al. (2008)
	NO, NO_2, NO_y	Gas-phase chemiluminescence	Pollack et al. (2010), Ryerson et al. (1999), Ryerson et al. (1998)
DC3 DC-8	isoprene, monoterpenes, benzene, toluene, xylene<br>methanol, acetone, acetaldehyde, MVK+MACR<br>acetonitrile	PTR-MS	Wisthaler et al. (2002)
	formaldehyde	DFGAS (base)	Richter et al. (2015), Weibring et al. (2010)
	formaldehyde	ISAF-LIF (sens)	Cazorla et al. (2015), DiGangi et al. (2011), Hottle et al. (2009)
	ISOPOOH+IEPOX, glycolaldehyde, hydroxyacetone, peroxyacetic acid, CH_3OOH<br>ISOPN, PROPNN	CIT-CIMS (CF_3O^-)	Crounse et al. (2006), St Clair et al. (2010)
	2-$BuONO_2$, 3-methyl-2-$BuONO_2$, 3-$PenONO_2$, 2-$PenONO_2$<br>C_2H_6, C_3H_8, i-butane, n-butane, i-pentane, n-pentane, 2,3-dimethylbutane, 2-methylpentane, 3-methylpentane, n-hexane, n-heptane, 2,4-dimethylpentane, 2-methylhexane, 2,3-dimethylpentane, 3-methylhexane, 2,2,4-trimethylpentane, C_2H_4, propene, trans-2-butene, cis-2-butene, cyclopentane, methylcyclopentane, cyclohexane, methylcyclohexane, C_2H_2	WAS	Blake et al. (2003)
	PAN, PPN	CIMS	Huey (2007), Kim et al. (2007), Slusher et al. (2004)
	MPN	TD-LIF	Wooldridge et al. (2010)
	NO, NO_2, NO_y	Gas-phase chemiluminescence	Pollack et al. (2010), Ryerson et al. (1999), Ryerson et al. (1998)
DC3 GV	i-butane, n-butane, i-pentane, n-pentane, n-hexane, n-heptane, benzene, toluene, ethylbenzene+m,p-xylene, o-xylene, isoprene, alpha-pinene, camphene, beta-pinene, limonene<br>methanol, ethanol, methyl butenol, acetone, MEK, propanal, butanal, acetaldehyde, MVK, MACR<br>acetonitrile	TOGA	Apel et al. (2010)
	formaldehyde	CAMS	Fried et al. (2011)
	CH_3OOH	PCIMS	O'Sullivan et al. (2018)
	NO, NO_2	Gas-phase chemiluminescence	Weinheimer et al. (1994)
SENEX	isoprene, monoterpenes, benzene, toluene, xylene<br>methanol, acetone, methyl ethyl ketone, acetaldehyde, MVK+MACR<br>acetonitrile	PTR-MS	de Gouw and Warneke (2007)
	C_2H_6, C_3H_8, i-butane, n-butane, i-pentane, n-pentane, n-hexane, C_2H_4, propene, C_2H_2	WAS	Lerner et al. (2017), Gilman et al. (2009)
	formaldehyde	ISAF-LIF	Cazorla et al. (2015), DiGangi et al. (2011), Hottle et al. (2009)
	ISOPOOH+IEPOX<br>ISOPN, MVKN+MACRN<br>HCOOH, $C_2H_4O_3$, $C_3H_4O_3$, $C_3H_4O_4$, $C_5H_8O_4$, $C_9H_{14}O_4$, $C_{10}H_{16}O_3$	UW CIMS (sens)	Lee et al. (2014)
	HCOOH	NOAA CIMS (base)	
	glyoxal	ACES	Min et al. (2016)

	PAN, PPN	CIMS	_ENREF_28, Zheng et al. (2011), Osthoff et al. (2008), Slusher et al. (2004)
	NO, NO_2, NO_y	Gas-phase chemiluminescence	Pollack et al. (2010), Ryerson et al. (1999), Ryerson et al. (1998)
$SEAC^4RS$	isoprene, monoterpenes, benzene, toluene<br>methanol, acetone, acetaldehyde, MVK+MACR<br>acetonitrile	PTR-MS	Wisthaler et al. (2002)
	formaldehyde	CAMS (base)	Fried et al. (2011)
	formaldehyde	ISAF-LIF (sens)	Cazorla et al. (2015), DiGangi et al. (2011), Hottle et al. (2009)
	ISOPOOH+IEPOX, hydroxyacetone, peroxyacetic acid<br>ISOPN, PROPNN, MVKN+MACRN<br>$C_4O_4H_7N$, $C_5O_5H_9N$, BUTENE_HN	CIT-CIMS (CF_3O^-)	Crounse et al. (2006), St Clair et al. (2010)
	2-BuONO$_2$, 3-methyl-2-BuONO$_2$, 3-PenONO$_2$, 2-PenONO$_2$<br>C_2H_6, C_3H_8, i-butane, n-butane, i-pentane, n-pentane, neopentane, n-hexane, 2,3-dimethylbutane, 2-methylpentane, 3-methylpentane, n-heptane, C_2H_4, propene, trans-2-butene, cis-2-butene, 1-butene, i-butene, 1-pentene, C_2H_2	WAS	Blake et al. (2003)
	PAN, PPN	PAN-CIMS	Huey (2007), Kim et al. (2007), Slusher et al. (2004)
	MPN	TD-LIF	Wooldridge et al. (2010)
	NO, NO_2, NO_y	Gas-phase chemiluminescence	Pollack et al. (2010), Ryerson et al. (1999), Ryerson et al. (1998)
DISCOVER-AQ	formaldehyde	DFGAS	Richter et al. (2015), Weibring et al. (2010)
	non-methane VOCs	PTR(-ToF-)MS	Müller et al. (2016), Müller et al. (2014)
	ethane (DISCOVER-AQ CO)	TILDAS	Yacovitch et al. (2014)
	NO, NO_2, NO_y	Gas-phase chemiluminescence	Weinheimer et al. (1994)
FRAPPÉ	formaldehyde, ethane	CAMS	Fried et al. (2011)
	monoterpenes, acetonitrile<br>methanol, acetaldehyde, acetone, isoprene, MVK+MACR, methyl ethyl ketone, benzene, toluene, C8 aromatics	PTR-MS (sens)	Kaser et al. (2013)
	methanol, acetaldehyde, acetone, isoprene, MVK, MACR, methyl ethyl ketone, benzene, toluene, ethylbenzene-m-p-xylene+o-xylene<br>ethanol, propanal, butanal, methyl butenol<br>C_3H_8, i-butane, n-butane, i-pentane, n-pentane, n-hexane,2-methylpentane, 3-methylpentane, n-heptane<br>tert-butylnitrate, 2-butylnitrate-n-butylnitrate, 2-pentylnitrate-3-pentylnitrate	TOGA (base)	Apel et al. (2010)
	CH_3OOH, HCOOH, Acetic acid	PCIMS	Treadaway et al. (2018)
	C_2H_4, propene, cyclopentane, methylcyclopentane, cyclohexane, methylcyclohexane, C_2H_2	WAS	Blake et al. (2003)
	PAN, PPN	PAN-CIMS	Zheng et al. (2011)
	NO, NO_2	Gas-phase chemiluminescence	(Weinheimer et al. (1994))

Table S2. Sensitivity of spatially aggregated model performance for total VOC-carbon to the use of data from alternate instruments for co-measured VOCs.

	Base case[a]	DC3 LIF HCHO	SEAC4RS LIF HCHO	SENEX UW CIMS HCOOH	FRAPPÉ PTRMS VOCs	All
NMB						
FT (>3km)	-63.7%	-62.0%	-63.8%	-63.7%	-63.7%	-62.1%
PBL (<2km)	-37.2%	-36.3%	-37.3%	-37.2%	-37.3%	-36.3%
R^2						
FT (>3km)	0.066	0.097	0.067	0.065	0.066	0.098
PBL (<2km)	0.361	0.445	0.362	0.369	0.360	0.449

[a]Base case uses DC3 DFGAS HCHO, SEAC4RS CAMS HCHO, SENEX NOAA CIMS HCOOH, FRAPPÉ TOGA methanol, acetaldehyde, acetone, isoprene, MVK, MACR, MEK, benzene, toluene, C8 aromatics.

Table S3. Sensitivity of spatially aggregated model performance for total VOC reactivity to the use of data from alternate instruments for co-measured VOCs.

	Base case[a]	DC3 LIF HCHO	SEAC4RS LIF HCHO	SENEX UW CIMS HCOOH	FRAPPÉ PTRMS VOCs	All
NMB						
FT (>3km)	-62.6%	-61.9%	-63.2%	-62.6%	-62.6%	-62.5%
PBL (<2km)	-33.9%	-32.7%	-34.0%	-33.7%	-33.9%	-32.8%
R^2						
FT (>3km)	0.043	0.045	0.046	0.043	0.043	0.048
PBL (<2km)	0.542	0.629	0.542	0.547	0.542	0.631

[a]Base case uses DC3 DFGAS HCHO, SEAC4RS CAMS HCHO, SENEX NOAA CIMS HCOOH, FRAPPÉ TOGA methanol, acetaldehyde, acetone, isoprene, MVK, MACR, MEK, benzene, toluene, C8 aromatics.

Table S4. Sensitivity of campaign-aggregated mixing ratio and model bias to the use of data from alternate instruments for co-measured VOCs

		PBL			FT		
		Mixing ratio (ppbC)	Model bias (ppbC)	Model bias (%)	Mixing ratio (ppbC)	Model bias (ppbC)	Model bias (%)
Formaldehyde	base	1.930	-0.401	-22.6	0.230	-0.048	-27.8
	sens	1.944	-0.424	-23.3	0.250	-0.072	-36.9
Formic acid	base	1.006	-0.721	-76.4	0.430	-0.330	-86.3
	sens	0.972	-0.709	-75.8	0.244	-0.153	-73.5
Methanol	base	4.238	-2.135	-59.9	1.299	-0.924	-77.7
	sens	4.191	-2.062	-58.8	1.303	-0.927	-77.7
Acetaldehyde	base	1.162	-0.545	-57.3	0.185	-0.156	-91.0
	sens	1.212	-0.580	-59.3	0.186	-0.159	-91.1
Acetone	base	6.671	-2.150	-34.7	3.459	-1.761	-52.3
	sens	6.803	-2.272	-36.0	3.466	-1.776	-52.4
Isoprene	base	0.259	-0.116	-74.1	0.019	-0.018	-100.0
	sens	0.309	-0.155	-78.1	0.020	-0.019	-100.0
MVK+MACR	base	0.424	-0.165	-58.5	0.035	-0.031	-99.7
	sens	0.450	-0.177	-58.5	0.035	-0.031	-99.7
MEK	base	0.636	0.240	43.4	0.167	-0.077	-75.5
	sens	0.697	0.187	34.9	0.168	-0.078	-76.4
Benzene	base	0.291	-0.095	-38.9	0.108	-0.073	-72.9
	sens	0.298	-0.105	-40.6	0.108	-0.074	-73.0
Toluene	base	0.210	0.041	13.6	0.026	-0.021	-97.8
	sens	0.221	0.039	8.6	0.026	-0.021	-97.8
Xylene	base	0.112	-0.039	-59.5	0.014	-0.013	-100.0
	sens	0.126	-0.052	-62.9	0.015	-0.015	-100.0

Table S5. Sensitivity of campaign-aggregated reactivity and model bias to the use of data from alternate instruments for co-measured VOCs

		PBL			FT		
		Reactivity (s^{-1})	Model bias (s^{-1})	Model bias (%)	Reactivity (s^{-1})	Model bias (s^{-1})	Model bias (%)
Formaldehyde	base	0.343	-0.071	-22.6	0.023	-0.004	-26.7
	sens	0.346	-0.076	-23.3	0.026	-0.007	-35.8
Formic acid	base	0.009	-0.006	-76.4	0.003	-0.002	-86.3
	sens	0.009	-0.006	-75.8	0.002	-0.001	-73.5
Methanol	base	0.080	-0.039	-59.2	0.010	-0.008	-77.2
	sens	0.080	-0.038	-58.2	0.010	-0.008	-77.2
Acetaldehyde	base	0.191	-0.087	-57.4	0.018	-0.015	-90.7
	sens	0.198	-0.093	-59.3	0.018	-0.016	-90.8
Acetone	base	0.008	-0.003	-34.6	0.002	-0.001	-52.1
	sens	0.008	-0.003	-36.0	0.002	-0.001	-52.3
Isoprene	base	0.111	-0.050	-74.1	0.006	-0.005	-100.0
	sens	0.130	-0.066	-78.1	0.006	-0.005	-100.0
MVK+MACR	base	0.052	-0.021	-58.5	0.003	-0.003	-99.7
	sens	0.055	-0.022	-58.2	0.003	-0.003	-99.7
MEK	base	0.004	0.001	40.6	0.001	-0.000	-72.3
	sens	0.005	0.001	34.7	0.001	-0.000	-73.2
Benzene	base	0.001	-0.000	-38.1	0.000	-0.000	-73.3
	sens	0.001	-0.000	-40.6	0.000	-0.000	-73.4
Toluene	base	0.004	0.001	13.7	0.000	-0.000	-97.7
	sens	0.004	0.001	8.6	0.000	-0.000	-97.8
Xylene	base	0.007	-0.002	-59.5	0.001	-0.000	-100.0
	sens	0.008	-0.003	-62.9	0.001	-0.001	-100.0

Table S6. Correlation between observed OVOCs and model-derived biogenic ($\mathcal{B}_{OVOC}$) and anthropogenic ($\mathcal{A}_{OVOC}$) contributions.

	$SEAC^4RS$	SENEX	DISCOVER-AQ TX	Other campaigns
Acetaldehyde	0.71	0.62	0.65	
Formaldehyde	0.71	0.71	0.63	
Acetone	0.87	0.70	0.68	
MEK	n/a	0.68	0.62	
PAA	0.85	n/a	n/a	
Methanol	0.55	0.73	0.53	
MVK	0.74	0.75	0.82	<0.50
MACR	0.75	0.75	0.78	
HCOOH	n/a	0.79	n/a	
Acetic acid	n/a	n/a	0.44	
Glyoxal	n/a	0.79	n/a	
Glycolaldehyde	n/a	n/a	0.63	
Hydroxyacetone	0.80	n/a	n/a	

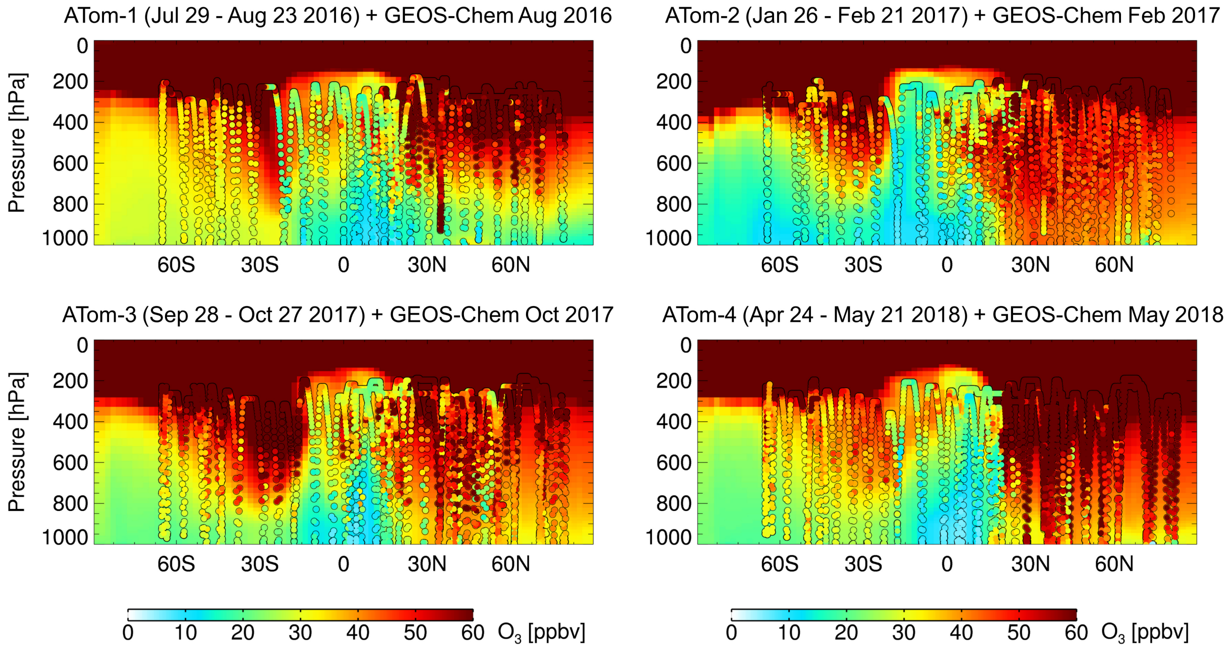

Figure S1. Ozone over the Pacific Ocean. Colored circles show airborne observations from the Atmospheric Tomography Mission (ATom) (Wofsy et al., 2018) within 100˚W-170˚E. Plotted in the background is the monthly mean ozone curtain simulated by GEOS-Chem (global simulation at 2˚×2.5˚) at ~177.5˚W for the corresponding month.

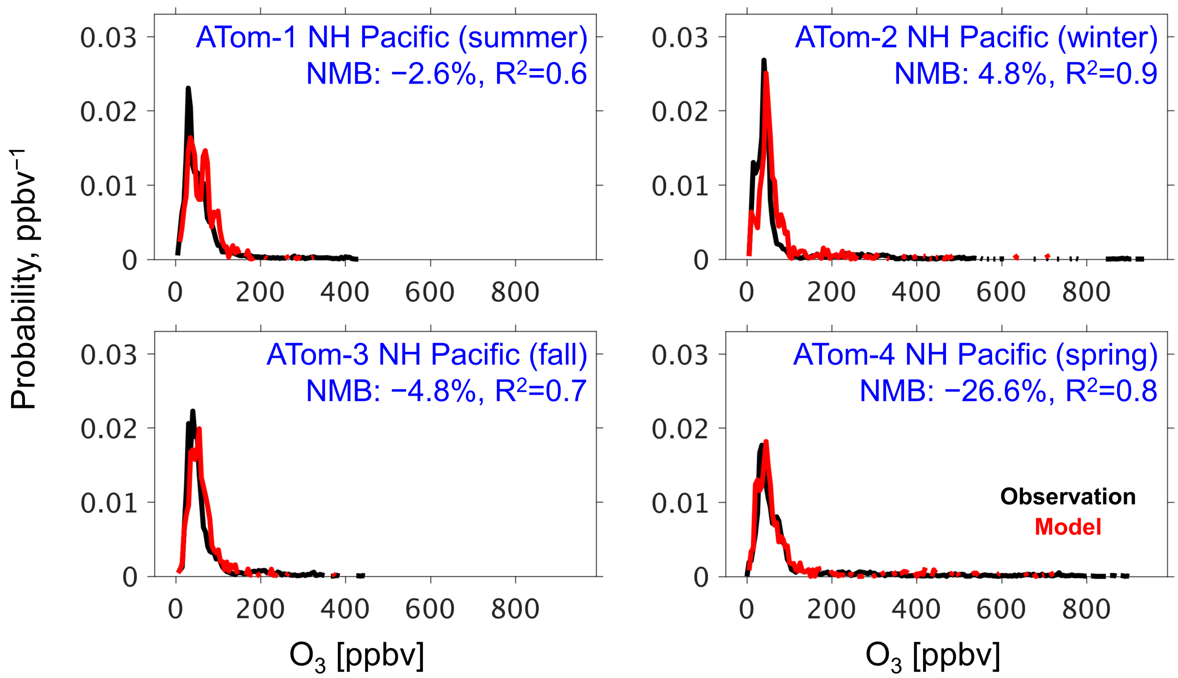

Figure S2. Ozone probability density functions over the northern Pacific (100˚W-170˚E, 0˚-90˚N). Plotted are ATom observations (black) and co-located GEOS-Chem model predictions (red), with correlations and normalized mean biases given inset.

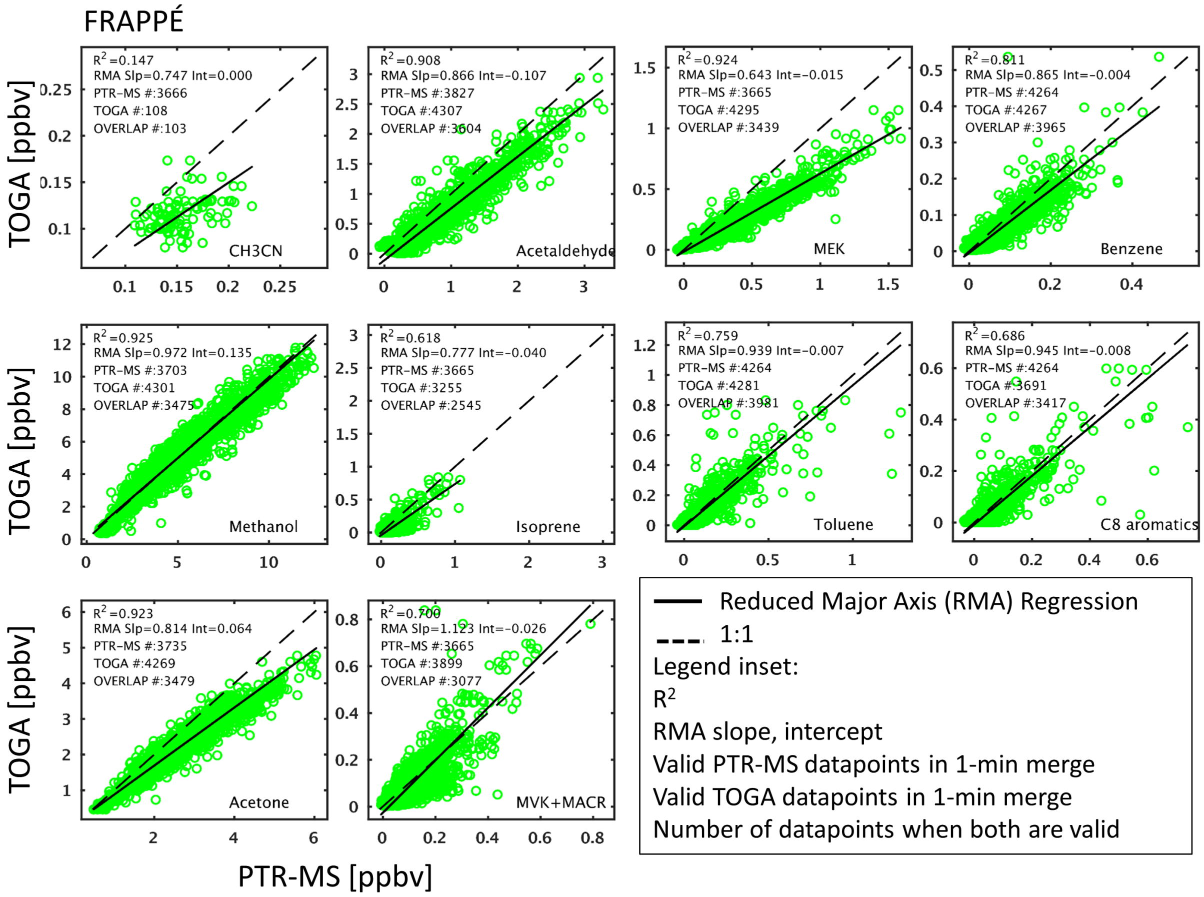

Figure S3. Inter-comparison of co-measured VOCs from FRAPPÉ.

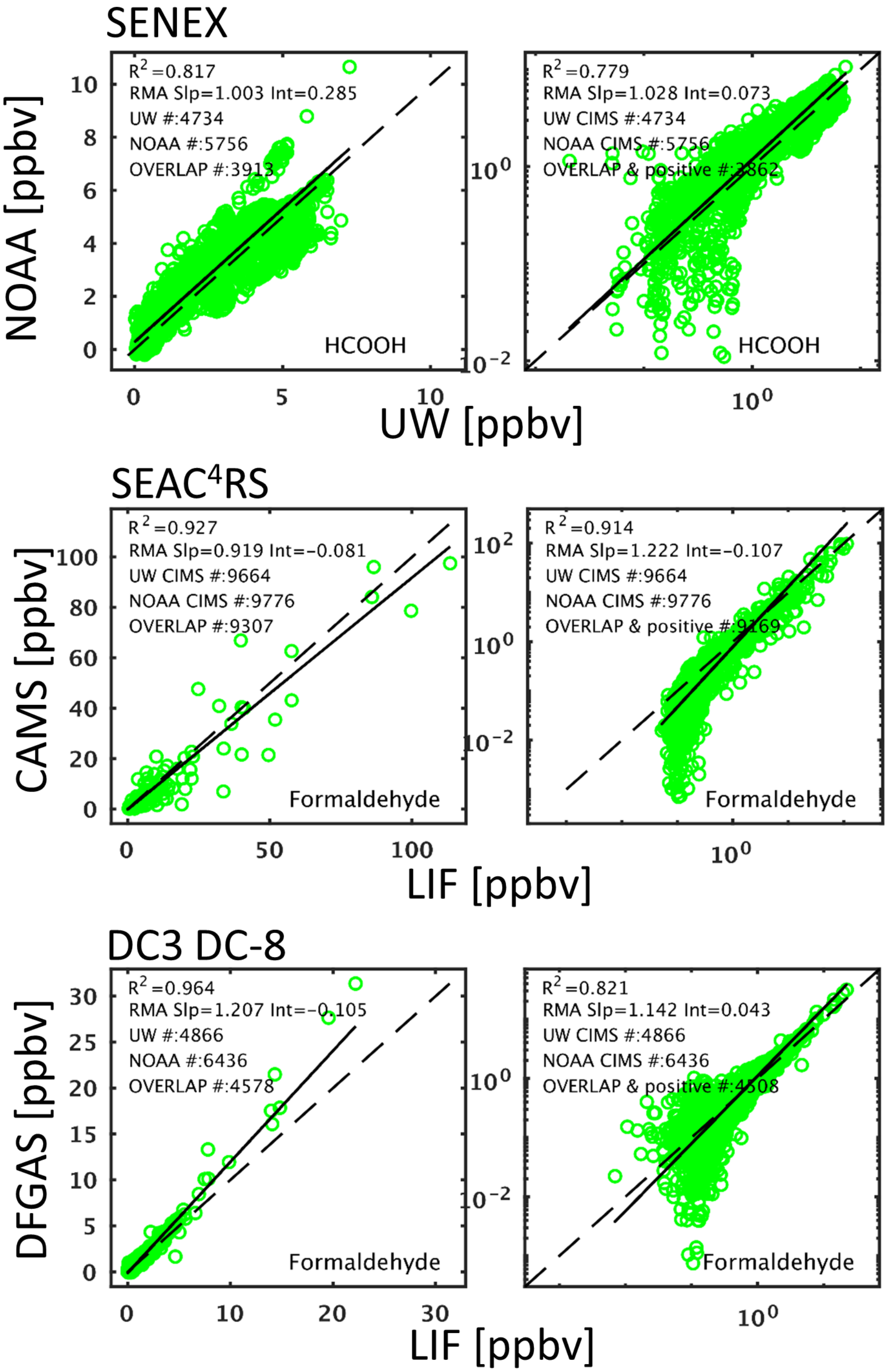

Figure S4. Inter-comparison of concurrent HCOOH measurements during SENEX and of concurrent formaldehyde measurements during DC3 DC-8 and SEAC^{4}RS. See legends in Figure S18.

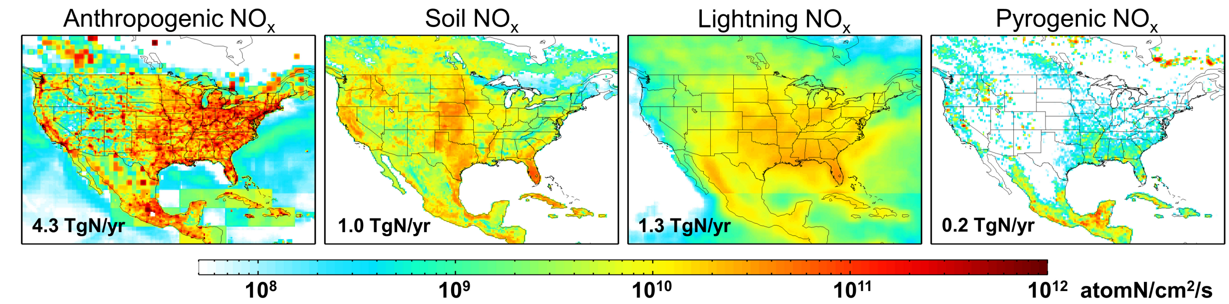

Figure S5. Annual NO_x emissions fluxes over North America as simulated by GEOS-Chem for 2013.

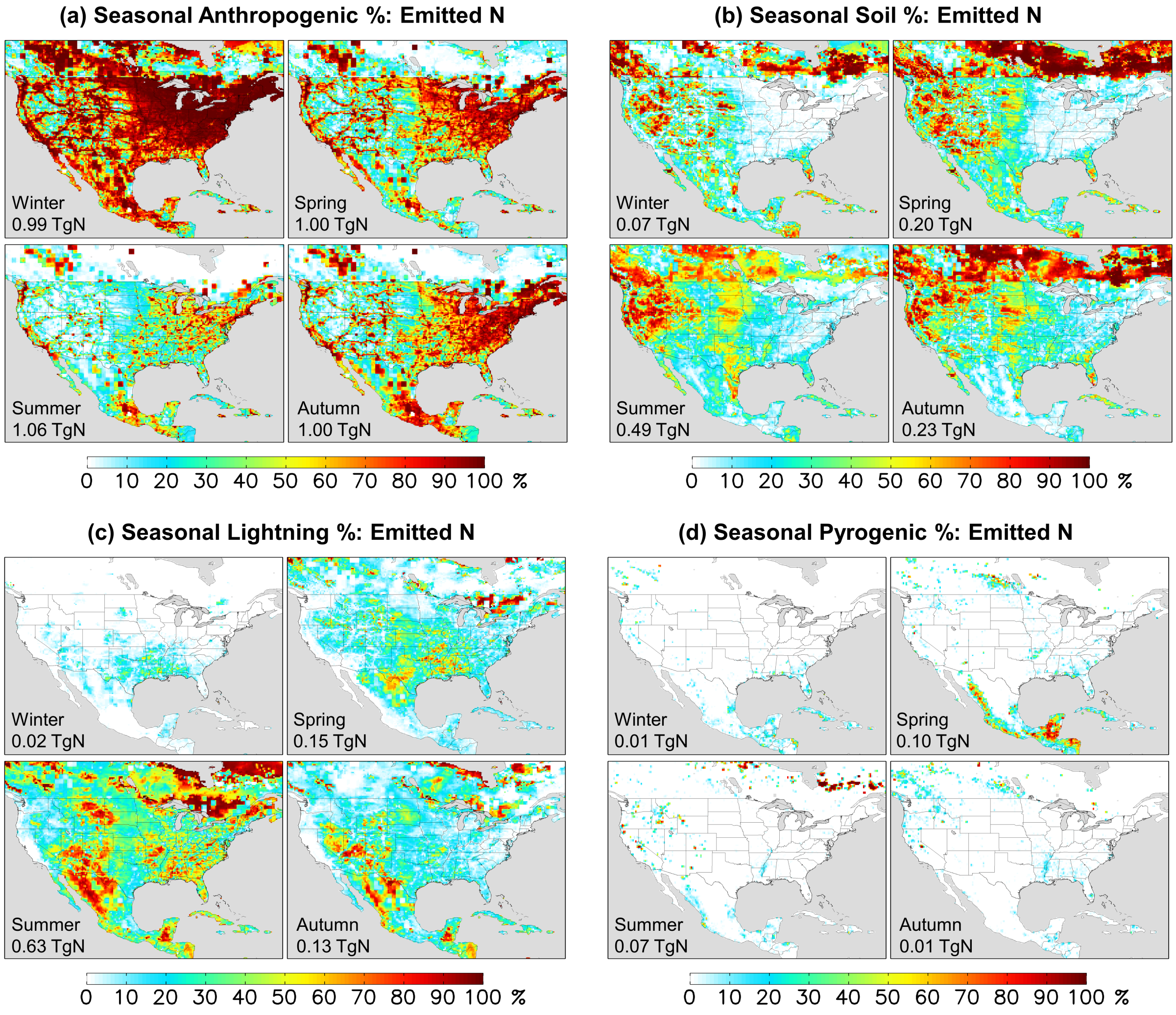

Figure S6. Seasonal contribution of each emission sector to total modeled NO_x emissions.

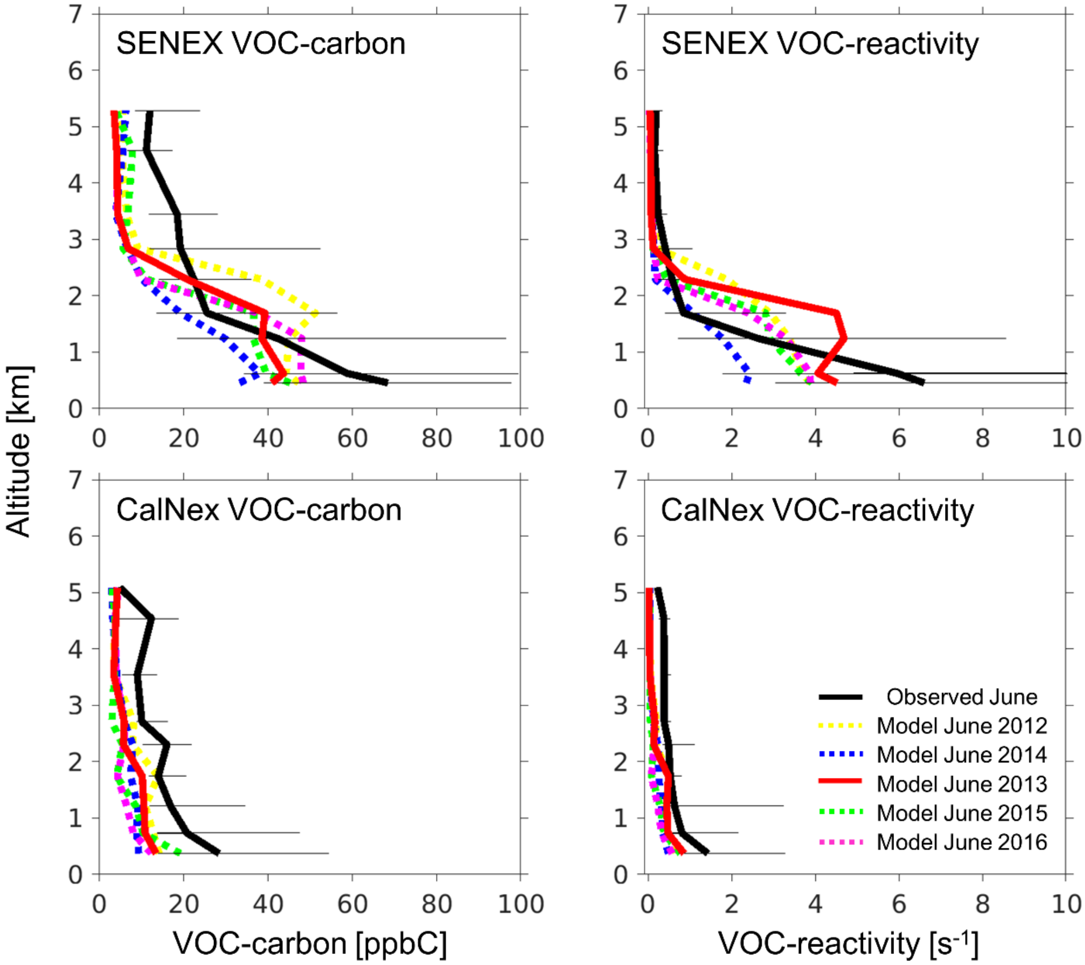

Figure S7. Vertical VOC-carbon and reactivity profiles as measured in 2013 and simulated by GEOS-Chem for June of 2012-2016 along the SENEX and CalNex aircraft flight tracks. Plotted are the observed (solid black lines) and predicted (2013: solid red lines; other years: dashed lines) median profiles, with horizontal bars indicating the 5th-95th percentiles measured for each vertical bin. Bin resolution is 0.5km below 3km and 1km above 3km. VOCs included in the profile correspond to the species shown in Fig. S17 (CalNex) and S18 (SENEX).

Given the range in measurement years spanned by the aircraft measurements, we performed a set of one-month simulations spanning 5 different years (June for 2012-2016) to assess the potential impact of interannual variability on these findings. Results are shown in Fig. S1 for the CalNex and SENEX flight tracks. In both cases the model-measurement VOC-C differences are highly consistent across years. We see a higher degree of interannual variability for VOC-reactivity over the SENEX domain, reflecting year-to-year differences in biogenic VOC emissions over this region. However, the key features of the comparison (a model underestimate near-surface, overly-flat vertical profile within the PBL, and strong FT underestimate) are consistent between our simulation year (2013) and the other four years.

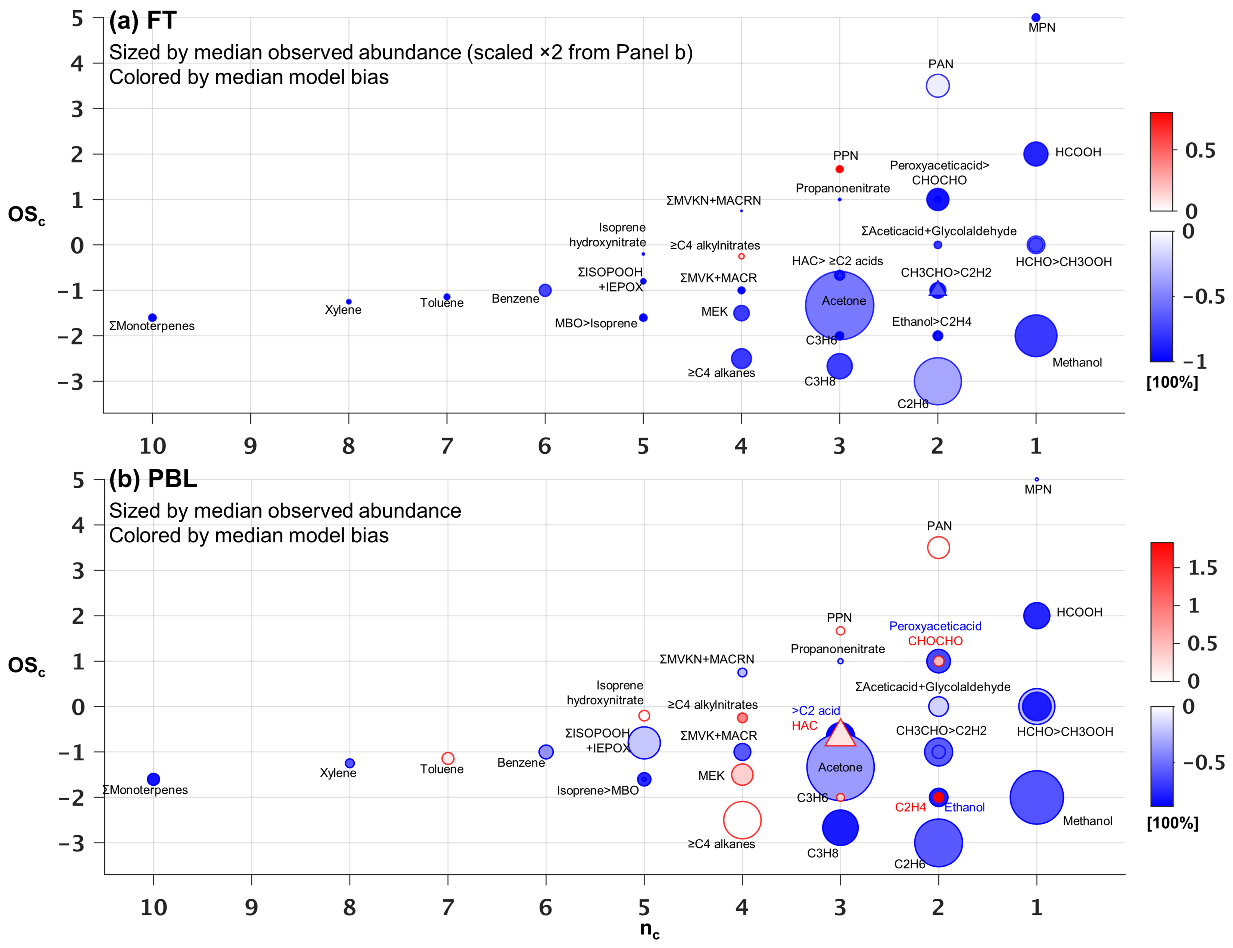

Figure S8. Same as Figure 5, but with the color shaded indicating the median relative model bias.

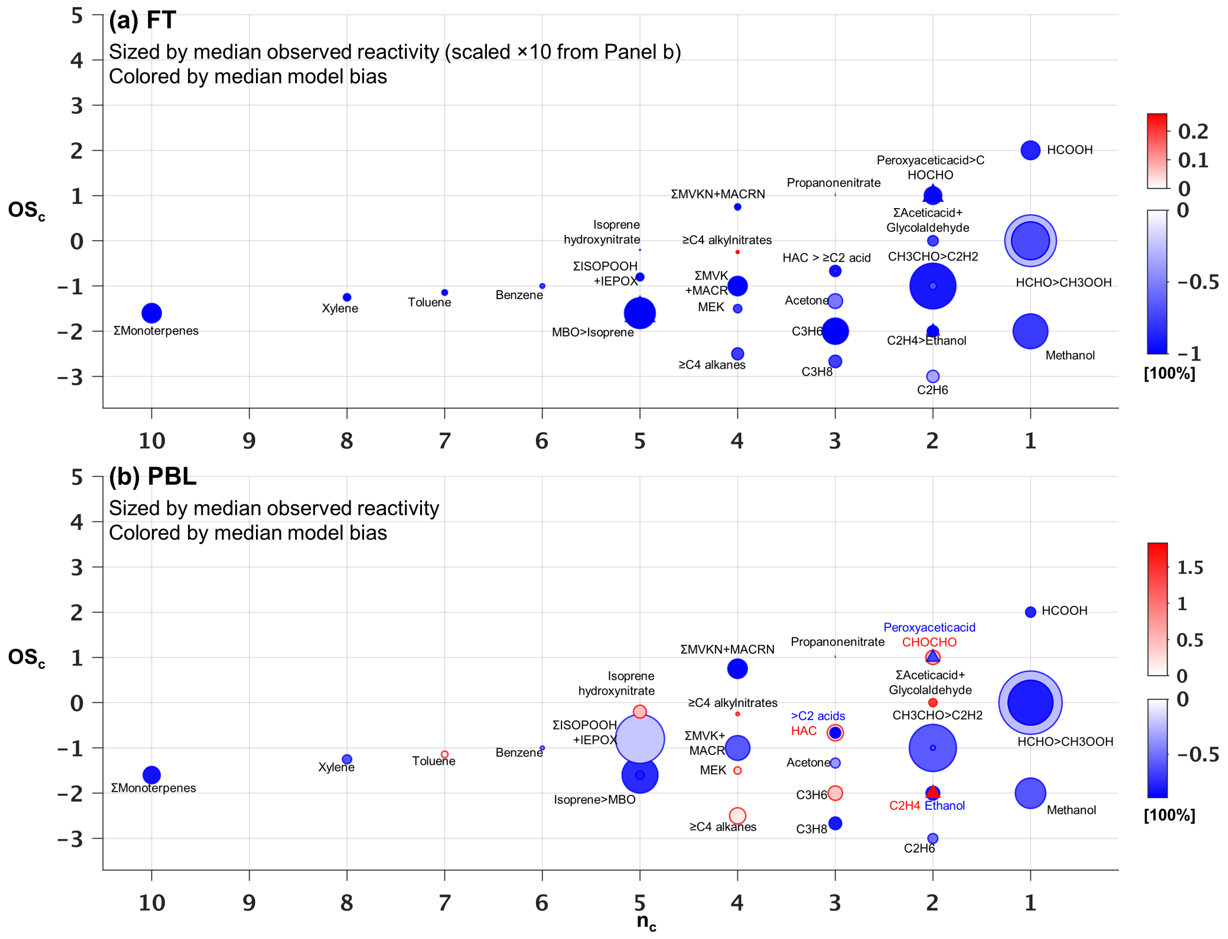

Figure S9. Same as Figure 6, but with the color shaded indicating the median relative model bias. Note that the relative model bias in VOC-carbon for a given species is identical to that for OH reactivity, except in the case of lumped or co-measured species.

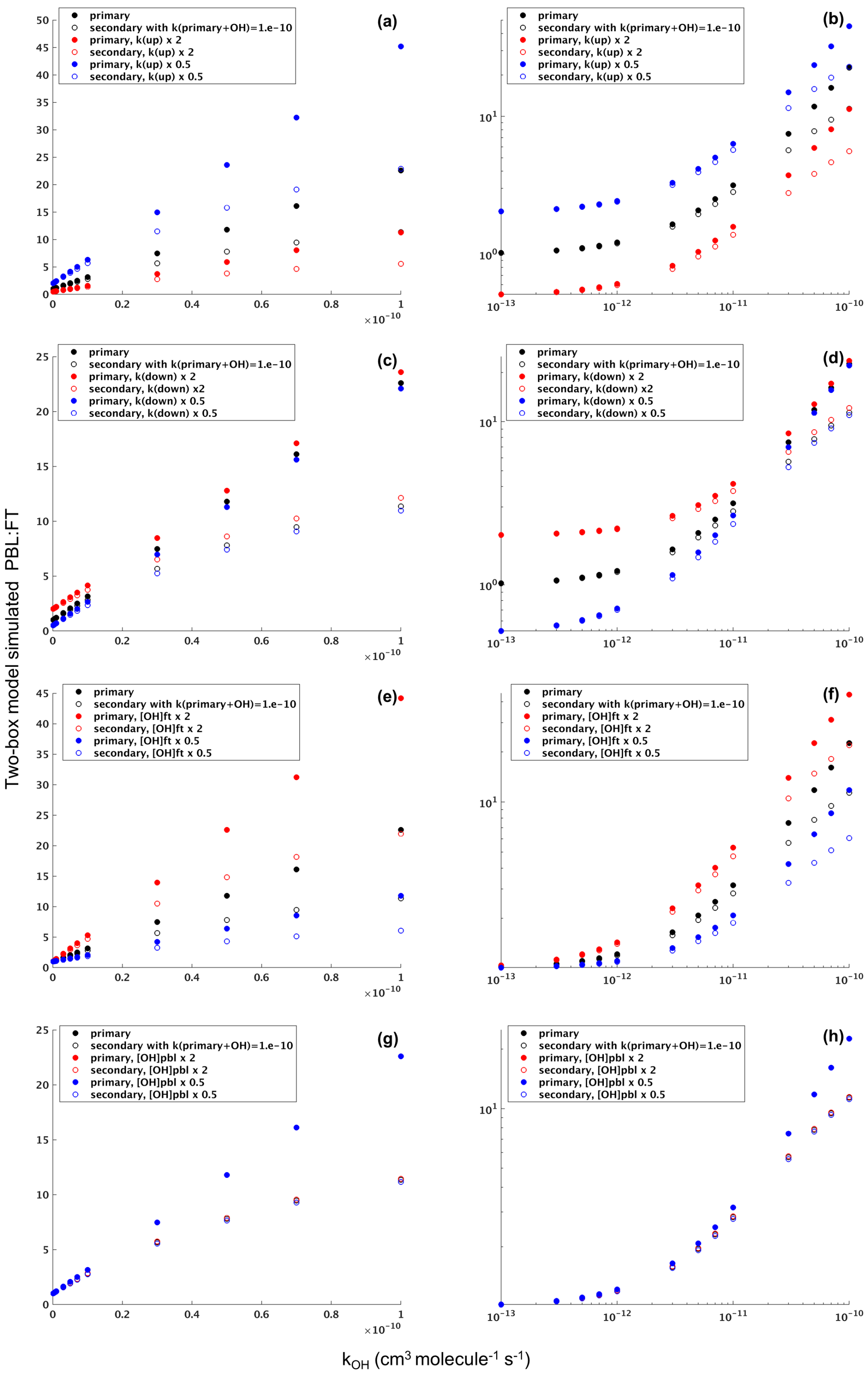

Figure S10. Sensitivity of the PBL:FT concentration ratio for primary and secondary VOCs to PBL-FT exchange rates, OH, and reactivity, based on a simple two-box model. See main text.

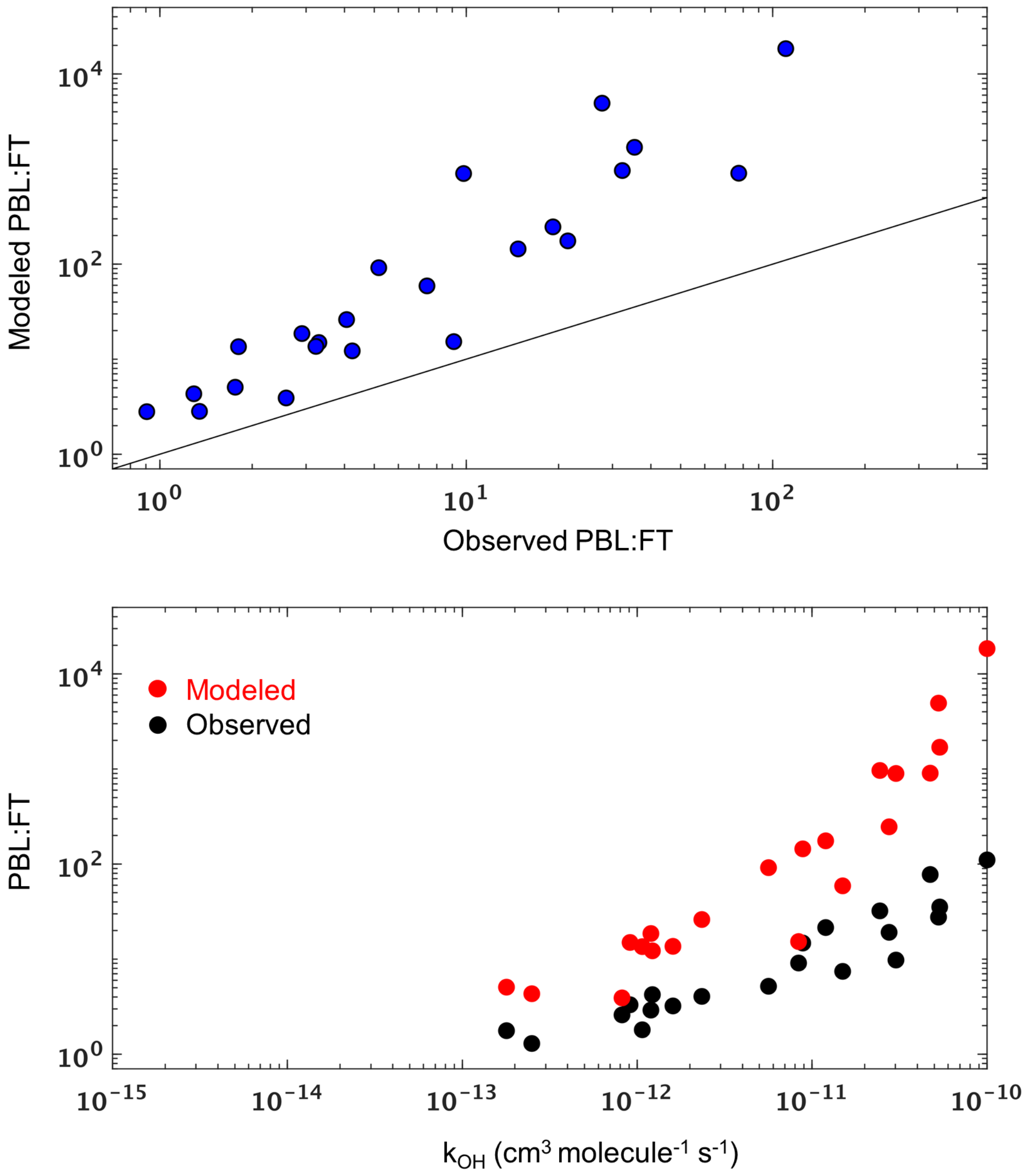

Figure S11. Same as Figure 9, but with model results from a sensitivity simulation with 40% reduced PBL depths.

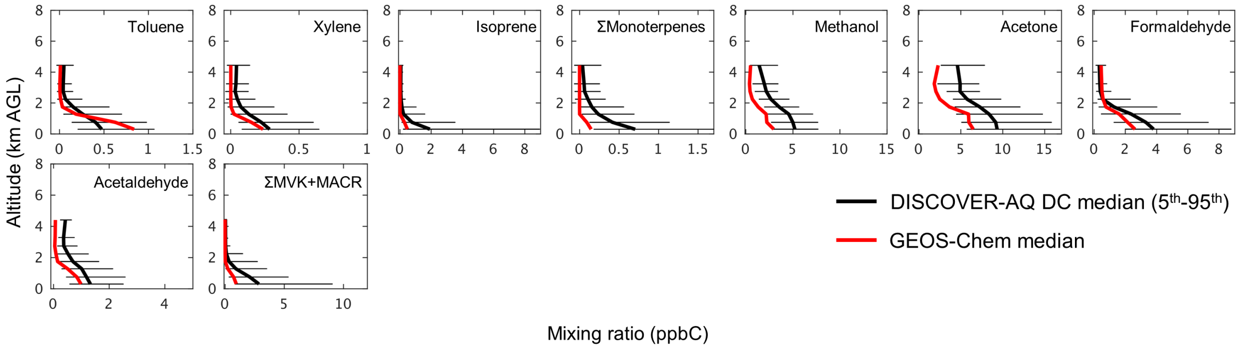

Figure S12. Vertical VOC profiles as measured and simulated by GEOS-Chem during the DISCOVER-AQ DC aircraft campaign. Plotted are the observed (black) and predicted (red) median profiles (in ppbC), with horizontal bars indicating the 5th - 95th percentiles measured for each bin. The vertical bin resolution is 0.5km below 3km and 1km above 3km. Fresh biomass burning and pollution plumes have been filtered out as described in-text.

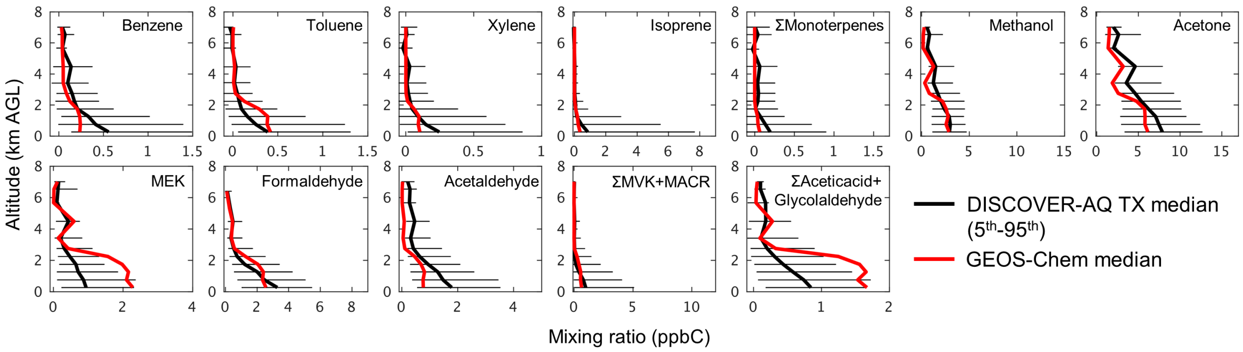

Figure S13. Same as Figure S5 but for the DISCOVER-AQ TX aircraft campaign.

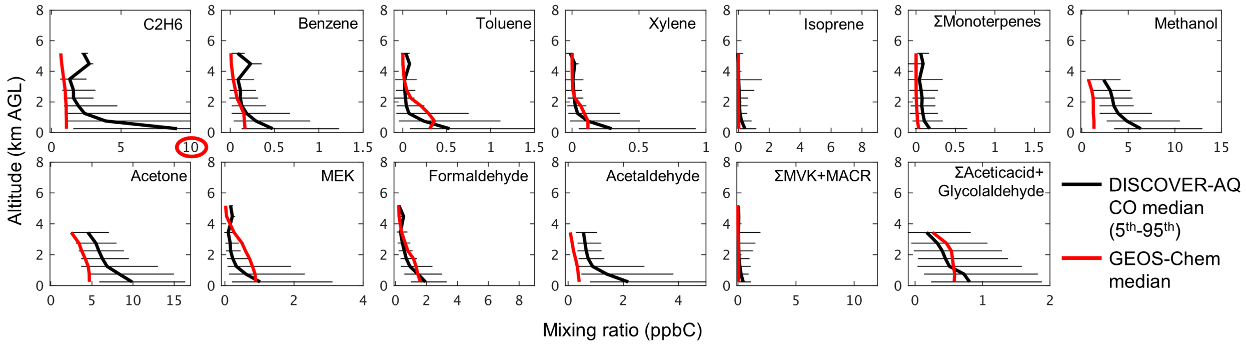

Figure S14. Same as Figure S5 but for the DISCOVER-AQ CO aircraft campaign. Red circles indicate axis scales that differ from others for the same compound in Fig. S5, 6, 9, 10.

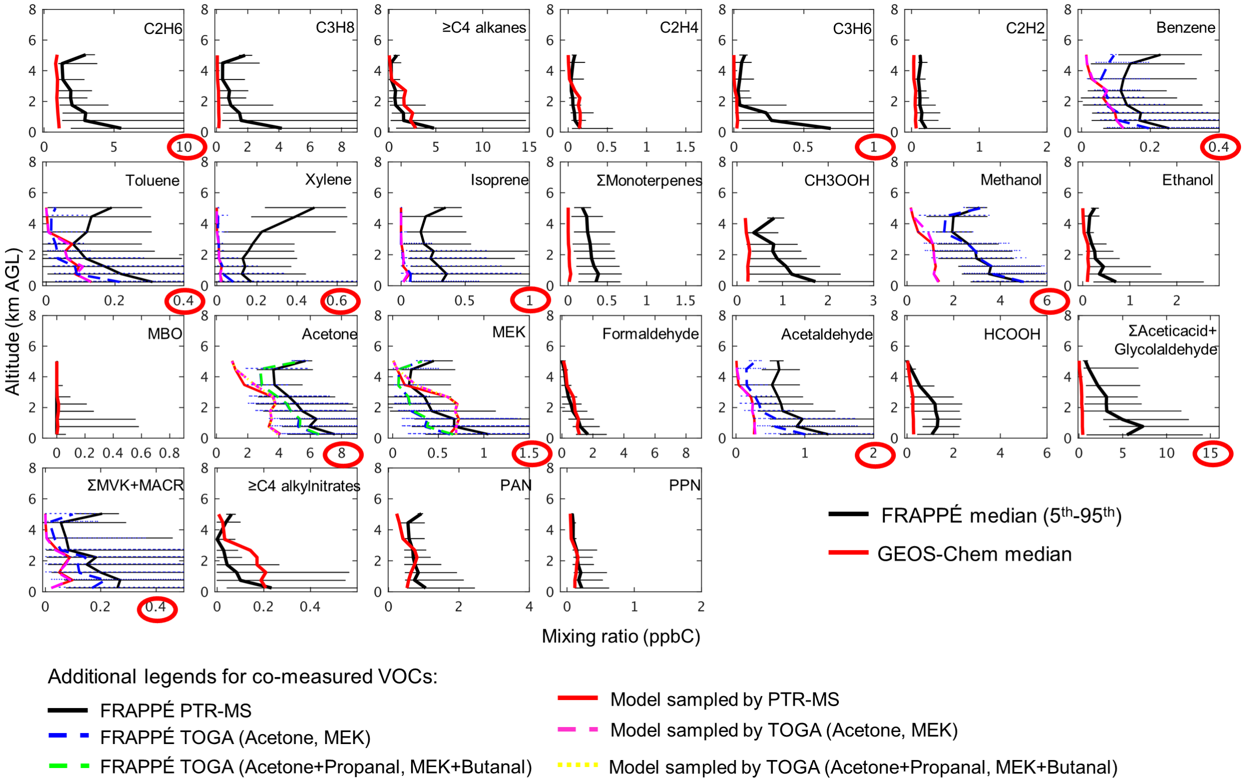

Figure S15. Same as Figure S5 but for the FRAPPÉ aircraft campaign. Red circles indicate axis scales that differ from others for the same compound in Fig. S5, 6, 9, 10.

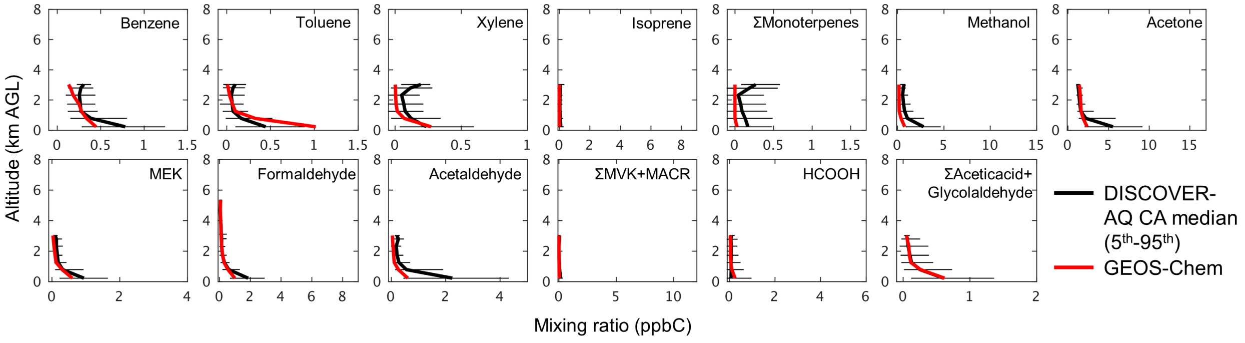

Figure S16. Same as Figure S5 but for the DISCOVER-AQ CA aircraft campaign.

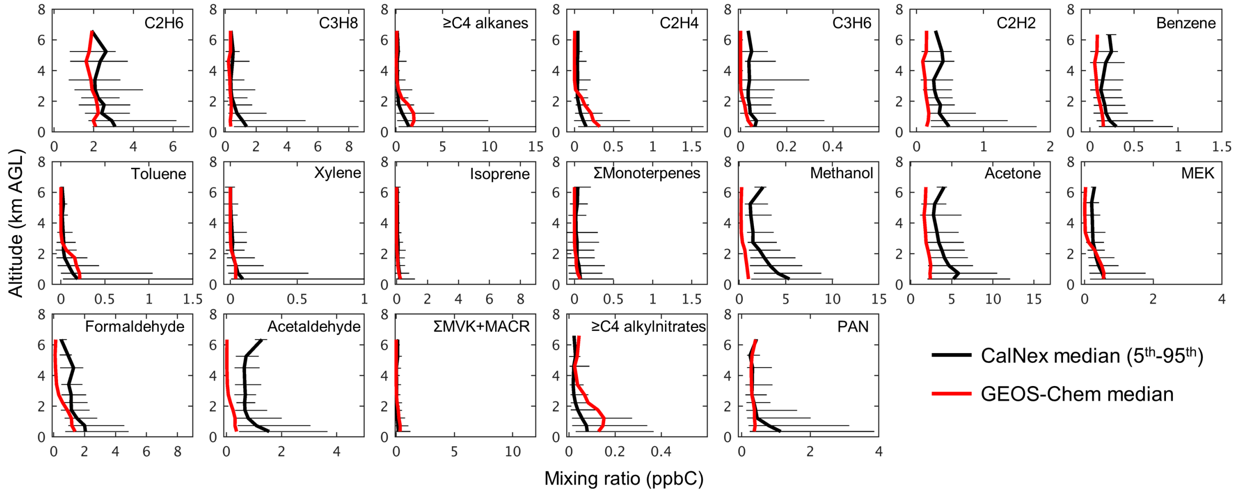

Figure S17. Same as Figure S5 but for the CalNex aircraft campaign.

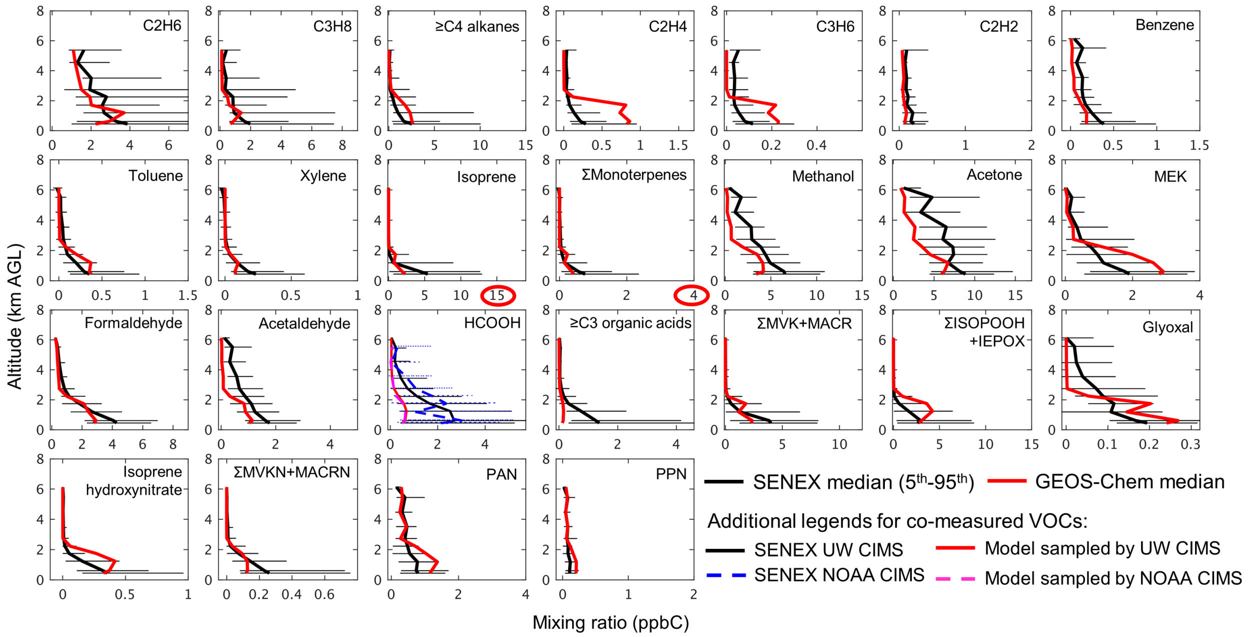

Figure S18. Same as Figure S5 but for the SENEX aircraft campaign. Red circles indicate axis scales that differ from others for the same compound in Figures S5, 6, 9, 10.

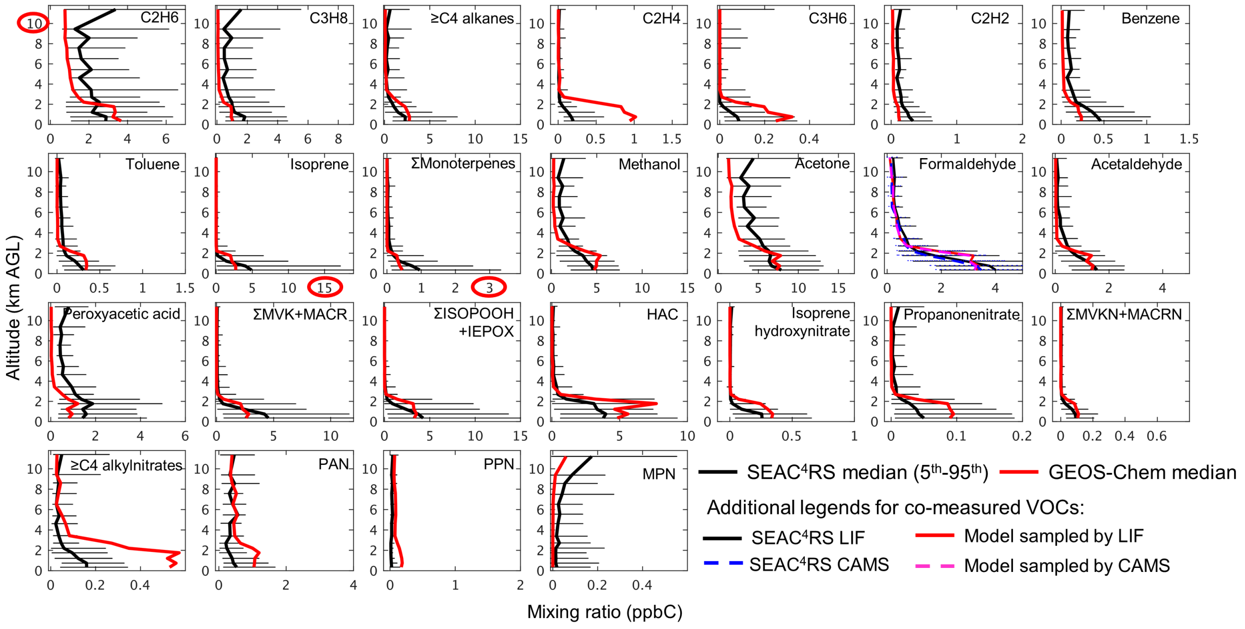

Figure S19. Same as Figure S5 but for the SEAC^{4}RS aircraft campaign. Red circles indicate axis scales that differ from others for the same compound in Figures S5, 6, 9, 10.

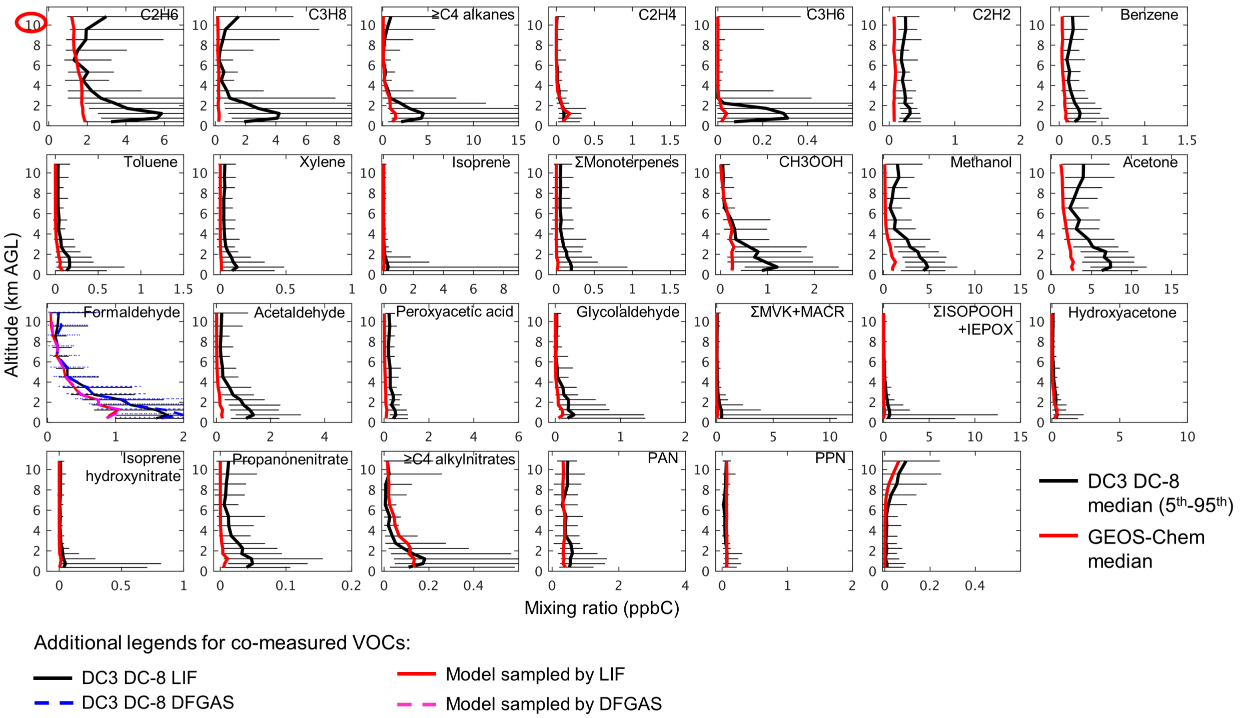

Figure S20. Same as figure S5 but for the DC3 DC-8 aircraft observation. Red circles indicate axis scales that differ from others for the same compound in Figures S5, 6, 9, 10.

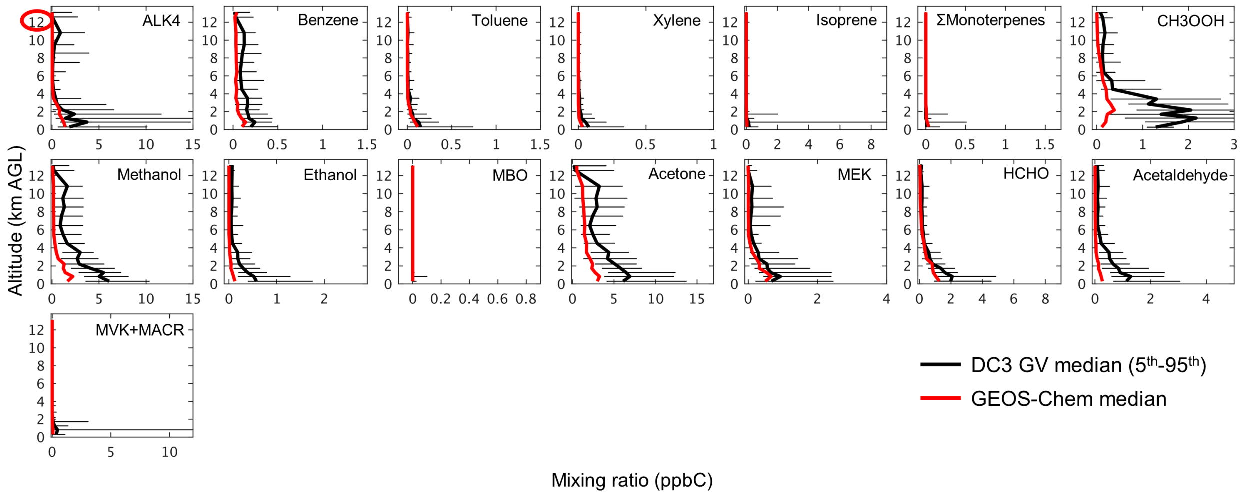

Figure S21. Same as figure S5 but for the DC3 GV aircraft observation. Red circles indicate axis scales that differ from others for the same compound in Figures S5, 6, 9, 10.

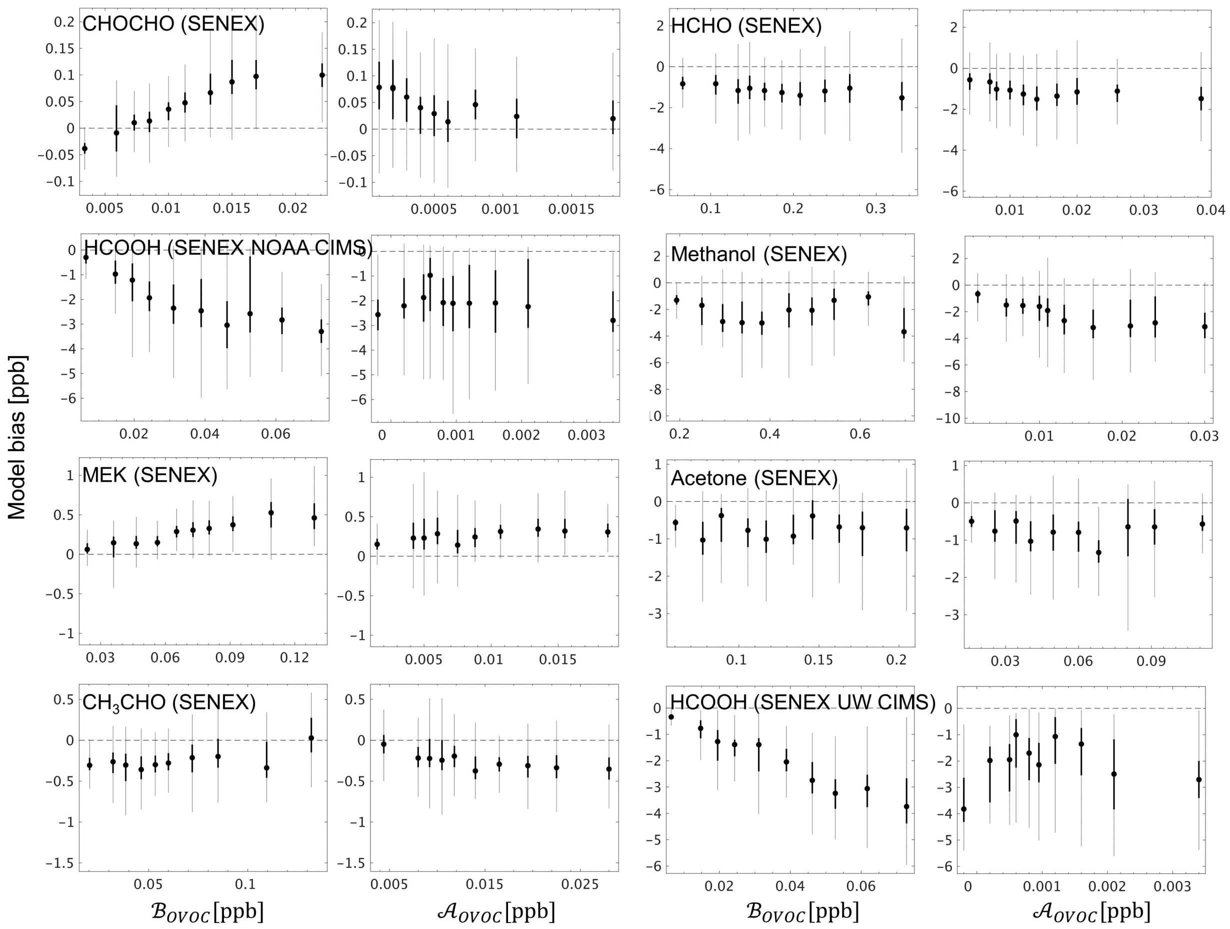

Figure S22. Same as Figure 10 but for the SENEX campaign.

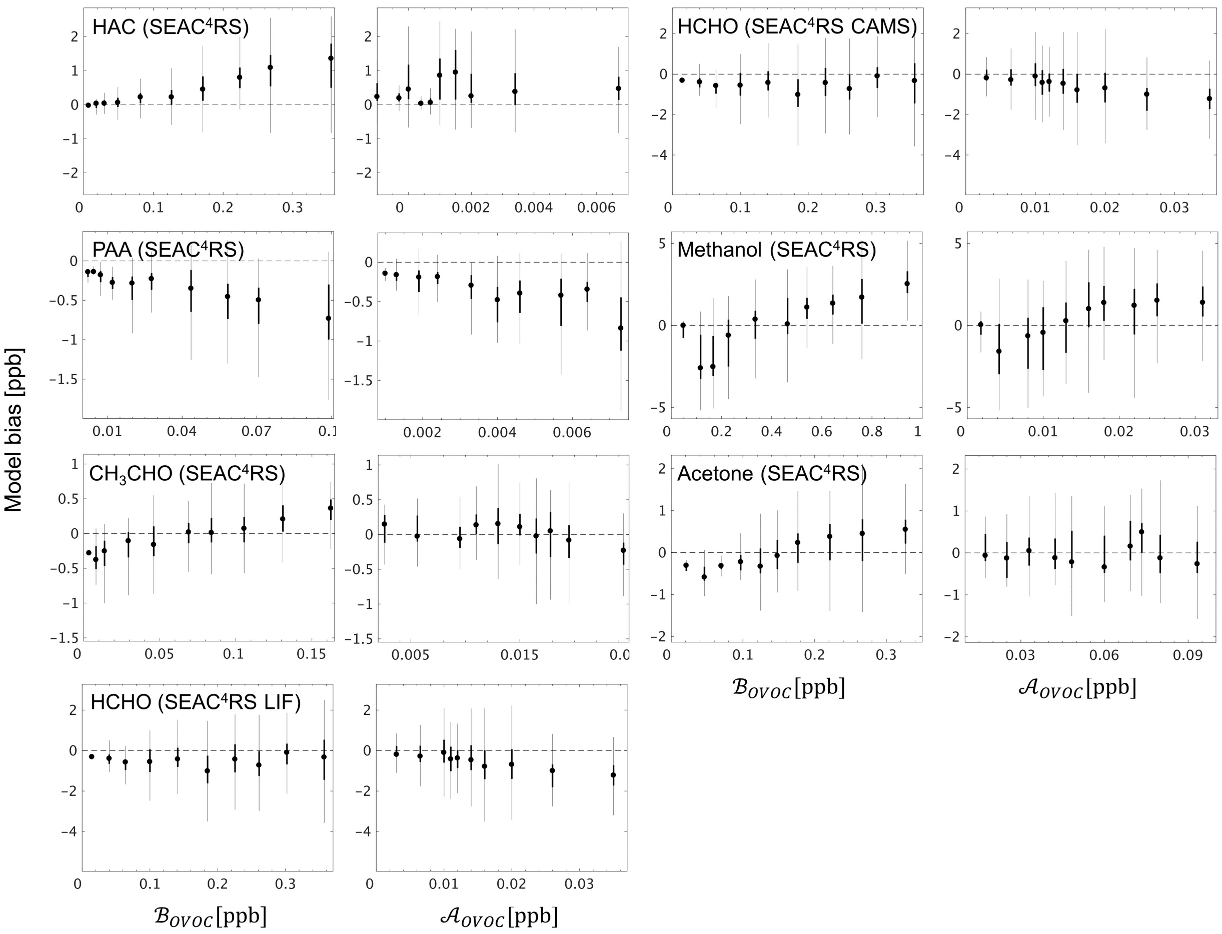

Figure S23. Same as Figure 10 but for the SEAC[4]RS campaign.

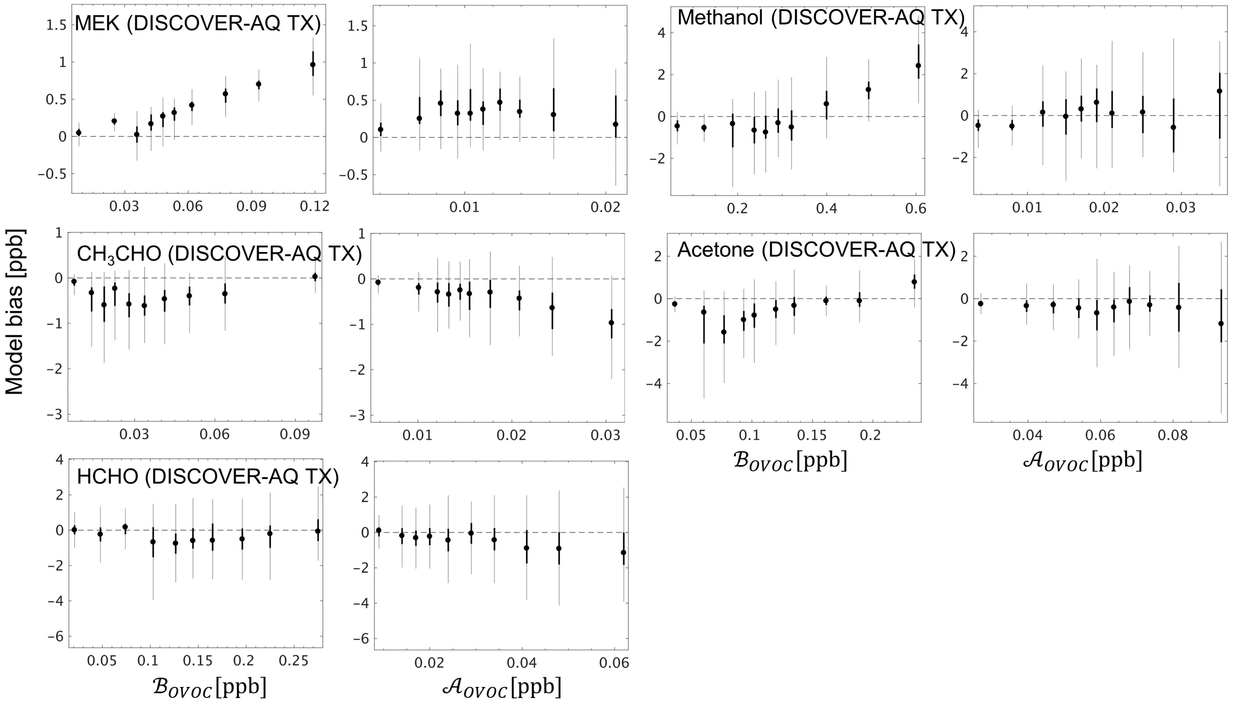

Figure S24. Same as Figure 10 but for the DISCOVER-AQ TX campaign.

References

Apel, E. C., Emmons, L. K., Karl, T., Flocke, F., Hills, A. J., Madronich, S., Lee-Taylor, J., Fried, A., Weibring, P., Walega, J., Richter, D., Tie, X., Mauldin, L., Campos, T., Weinheimer, A., Knapp, D., Sive, B., Kleinman, L., Springston, S., Zaveri, R., Ortega, J., Voss, P., Blake, D., Baker, A., Warneke, C., Welsh-Bon, D., de Gouw, J., Zheng, J., Zhang, R., Rudolph, J., Junkermann, W., and Riemer, D. D.: Chemical evolution of volatile organic compounds in the outflow of the Mexico City Metropolitan area, Atmos. Chem. Phys., 10, 2353-2375, https://doi.org/10.5194/acp-10-2353-2010, 2010.

Blake, N. J., Blake, D. R., Swanson, A. L., Atlas, E., Flocke, F., and Rowland, F. S.: Latitudinal, vertical, and seasonal variations of C1-C4 alkyl nitrates in the troposphere over the Pacific Ocean during PEM-Tropics A and B: Oceanic and continental sources, J. Geophys. Res. Atmos., 108, https://doi.org/10.1029/2001jd001444, 2003.

Cazorla, M., Wolfe, G. M., Bailey, S. A., Swanson, A. K., Arkinson, H. L., and Hanisco, T. F.: A new airborne laser-induced fluorescence instrument for in situ detection of formaldehyde throughout the troposphere and lower stratosphere, Atmos. Meas. Tech., 8, 541-552, https://doi.org/10.5194/amt-8-541-2015, 2015.

Colman, J. J., Swanson, A. L., Meinardi, S., Sive, B. C., Blake, D. R., and Rowland, F. S.: Description of the analysis of a wide range of volatile organic compounds in whole air samples collected during PEM-tropics A and B, Anal. Chem., 73, 3723-3731, https://doi.org/10.1021/ac010027g, 2001.

Crounse, J. D., McKinney, K. A., Kwan, A. J., and Wennberg, P. O.: Measurement of gas-phase hydroperoxides by chemical ionization mass spectrometry, Anal. Chem., 78, 6726-6732, https://doi.org/10.1021/ac0604235, 2006.

de Gouw, J., and Warneke, C.: Measurements of volatile organic compounds in the earth's atmosphere using proton-transfer-reaction mass spectrometry, Mass Spectrom. Rev., 26, 223-257, https://doi.org/10.1002/mas.20119, 2007.

DiGangi, J. P., Boyle, E. S., Karl, T., Harley, P., Turnipseed, A., Kim, S., Cantrell, C., Maudlin, R. L., Zheng, W., Flocke, F., Hall, S. R., Ullmann, K., Nakashima, Y., Paul, J. B., Wolfe, G. M., Desai, A. R., Kajii, Y., Guenther, A., and Keutsch, F. N.: First direct measurements of formaldehyde flux via eddy covariance: implications for missing in-canopy formaldehyde sources, Atmos. Chem. Phys., 11, 10565-10578, https://doi.org/10.5194/acp-11-10565-2011, 2011.

Fried, A., Cantrell, C., Olson, J., Crawford, J. H., Weibring, P., Walega, J., Richter, D., Junkermann, W., Volkamer, R., Sinreich, R., Heikes, B. G., O'Sullivan, D., Blake, D. R., Blake, N., Meinardi, S., Apel, E., Weinheimer, A., Knapp, D., Perring, A., Cohen, R. C., Fuelberg, H., Shetter, R. E., Hall, S. R., Ullmann, K., Brune, W. H., Mao, J., Ren, X., Huey, L. G., Singh, H. B., Hair, J. W., Riemer, D., Diskin, G., and Sachse, G.: Detailed comparisons of airborne formaldehyde measurements with box models during the 2006 INTEX-B and MILAGRO campaigns: potential evidence for significant impacts of unmeasured and multi-generation volatile organic carbon compounds, Atmos. Chem. Phys., 11, 11867-11894, https://doi.org/10.5194/acp-11-11867-2011, 2011.

Gilman, J. B., Kuster, W. C., Goldan, P. D., Herndon, S. C., Zahniser, M. S., Tucker, S. C., Brewer, W. A., Lerner, B. M., Williams, E. J., Harley, R. A., Fehsenfeld, F. C., Warneke, C., and de Gouw, J. A.: Measurements of volatile organic compounds during the 2006 TexAQS/GoMACCS campaign: Industrial influences, regional characteristics, and diurnal dependencies of the OH reactivity, J. Geophys. Res. Atmos., 114, https://doi.org/10.1029/2008jd011525, 2009.

Hottle, J. R., Huisman, A. J., DiGangi, J. P., Kammrath, A., Galloway, M. M., Coens, K. L., and Keutsch, F. N.: A laser induced fluorescence-based instrument for in-situ measurements of atmospheric formaldehyde, Environ. Sci. Technol., 43, 790-795, https://doi.org/10.1021/es801621f, 2009.

Huey, L. G.: Measurement of trace atmospheric species by chemical ionization mass spectrometry: speciation of reactive nitrogen and future directions, Mass Spectrom. Rev., 26, 166-184, https://doi.org/10.1002/mas.20118, 2007.

Kaser, L., Karl, T., Schnitzhofer, R., Graus, M., Herdlinger-Blatt, I. S., DiGangi, J. P., Sive, B., Turnipseed, A., Hornbrook, R. S., Zheng, W., Flocke, F. M., Guenther, A., Keutsch, F. N., Apel, E., and Hansel, A.: Comparison of different real time VOC measurement techniques in a ponderosa pine forest, Atmos. Chem. Phys., 13, 2893-2906, https://doi.org/10.5194/acp-13-2893-2013, 2013.

Kim, S., Huey, L. G., Stickel, R. E., Tanner, D. J., Crawford, J. H., Olson, J. R., Chen, G., Brune, W. H., Ren, X., Lesher, R., Wooldridge, P. J., Bertram, T. H., Perring, A., Cohen, R. C., Lefer, B. L., Shetter, R. E., Avery, M., Diskin, G., and Sokolik, I.: Measurement of HO2NO2 in the free troposphere during the intercontinental chemical transport experiment - North America 2004, J. Geophys. Res. Atmos., 112, https://doi.org/10.1029/2006jd007676, 2007.

Lee, B. H., Lopez-Hilfiker, F. D., Mohr, C., Kurten, T., Worsnop, D. R., and Thornton, J. A.: An iodide-adduct high-resolution time-of-flight chemical-ionization mass spectrometer: application to atmospheric inorganic and organic compounds, Environ. Sci. Technol., 48, 6309-6317, https://doi.org/10.1021/es500362a, 2014.

Lerner, B. M., Gilman, J. B., Aikin, K. C., Atlas, E. L., Goldan, P. D., Graus, M., Hendershot, R., Isaacman-VanWertz, G. A., Koss, A., Kuster, W. C., Lueb, R. A., McLaughlin, R. J., Peischl, J., Sueper, D., Ryerson, T. B., Tokarek, T. W., Warneke, C., Yuan, B., and de Gouw, J. A.: An improved, automated whole air sampler and gas chromatography mass spectrometry analysis system for volatile organic compounds in the atmosphere, Atmos. Meas. Tech., 10, 291-313, https://doi.org/10.5194/amt-10-291-2017, 2017.

Min, K. E., Washenfelder, R. A., Dube, W. P., Langford, A. O., Edwards, P. M., Zarzana, K. J., Stutz, J., Lu, K., Rohrer, F., Zhang, Y., and Brown, S. S.: A broadband cavity enhanced absorption spectrometer for aircraft measurements of glyoxal, methylglyoxal, nitrous acid, nitrogen dioxide, and water vapor, Atmos. Meas. Tech., 9, 423-440, https://doi.org/10.5194/amt-9-423-2016, 2016.

Müller, M., Mikoviny, T., Feil, S., Haidacher, S., Hanel, G., Hartungen, E., Jordan, A., Mark, L., Mutschlechner, P., Schottkowsky, R., Sulzer, P., Crawford, J. H., and Wisthaler, A.: A compact PTR-ToF-MS instrument for airborne measurements of volatile organic compounds at high spatiotemporal resolution, Atmos. Meas. Tech., 7, 3763-3772, https://doi.org/10.5194/amt-7-3763-2014, 2014.

Müller, M., Anderson, B. E., Beyersdorf, A. J., Crawford, J. H., Diskin, G. S., Eichler, P., Fried, A., Keutsch, F. N., Mikoviny, T., Thornhill, K. L., Walega, J. G., Weinheimer, A. J., Yang, M., Yokelson, R. J., and Wisthaler, A.: In situ measurements and modeling of reactive trace gases in a small biomass burning plume, Atmos. Chem. Phys., 16, 3813-3824, https://doi.org/10.5194/acp-16-3813-2016, 2016.

O'Sullivan, D. W., Silwal, I. K. C., McNeill, A. S., Treadaway, V., and Heikes, B. G.: Quantification of gas phase hydrogen peroxide and methyl peroxide in ambient air: Using atmospheric pressure chemical ionization mass spectrometry with O2−, and O2−(CO2) reagent ions, Int. J. Mass Spectrom., 424, 16-26, https://doi.org/10.1016/j.ijms.2017.11.015, 2018.

Osthoff, H. D., Roberts, J. M., Ravishankara, A. R., Williams, E. J., Lerner, B. M., Sommariva, R., Bates, T. S., Coffman, D., Quinn, P. K., Dibb, J. E., Stark, H., Burkholder, J. B., Talukdar, R. K., Meagher, J., Fehsenfeld, F. C., and Brown, S. S.: High levels of nitryl chloride in the polluted subtropical marine boundary layer, Nat Geosci, 1, 324-328, https://doi.org/10.1038/ngeo177, 2008.

Pollack, I. B., Lerner, B. M., and Ryerson, T. B.: Evaluation of ultraviolet light-emitting diodes for detection of atmospheric NO2 by photolysis - chemiluminescence, J. Atmos. Chem., 65, 111-125, https://doi.org/10.1007/s10874-011-9184-3, 2010.

Richter, D., Weibring, P., Walega, J. G., Fried, A., Spuler, S. M., and Taubman, M. S.: Compact highly sensitive multi-species airborne mid-IR spectrometer, Appl. Phys. B: Lasers Opt., 119, 119-131, https://doi.org/10.1007/s00340-015-6038-8, 2015.

Ryerson, T. B., Buhr, M. P., Frost, G. J., Goldan, P. D., Holloway, J. S., Hubler, G., Jobson, B. T., Kuster, W. C., McKeen, S. A., Parrish, D. D., Roberts, J. M., Sueper, D. T., Trainer, M., Williams, J., and Fehsenfeld, F. C.: Emissions lifetimes and ozone formation in power plant plumes, J. Geophys. Res. Atmos., 103, 22569-22583, https://doi.org/10.1029/98jd01620, 1998.

Ryerson, T. B., Huey, L. G., Knapp, K., Neuman, J. A., Parrish, D. D., Sueper, D. T., and Fehsenfeld, F. C.: Design and initial characterization of an inlet for gas-phase NOy measurements from aircraft, J. Geophys. Res. Atmos., 104, 5483-5492, https://doi.org/10.1029/1998jd100087, 1999.

Schauffler, S. M., Atlas, E. L., Donnelly, S. G., Andrews, A., Montzka, S. A., Elkins, J. W., Hurst, D. F., Romashkin, P. A., Dutton, G. S., and Stroud, V.: Chlorine budget and partitioning during the Stratospheric Aerosol and Gas Experiment (SAGE) III Ozone Loss and Validation Experiment (SOLVE), J. Geophys. Res. Atmos., 108, https://doi.org/10.1029/2001jd002040, 2003.

Slusher, D. L., Huey, L. G., Tanner, D. J., Flocke, F. M., and Roberts, J. M.: A thermal dissociation-chemical ionization mass spectrometry (TD-CIMS) technique for the simultaneous measurement of peroxyacyl nitrates and dinitrogen pentoxide, J. Geophys. Res. Atmos., 109, https://doi.org/10.1029/2004jd004670, 2004.

St Clair, J. M., McCabe, D. C., Crounse, J. D., Steiner, U., and Wennberg, P. O.: Chemical ionization tandem mass spectrometer for the in situ measurement of methyl hydrogen peroxide, Rev. Sci. Instrum., 81, 094102, https://doi.org/10.1063/1.3480552, 2010.

Treadaway, V., Heikes, B. G., McNeill, A. S., Silwal, I. K. C., and O'Sullivan, D. W.: Measurement of formic acid, acetic acid and hydroxyacetaldehyde, hydrogen peroxide, and methyl peroxide in air by chemical ionization mass spectrometry: airborne method development, Atmos. Meas. Tech., 11, 1901-1920, https://doi.org/10.5194/amt-11-1901-2018, 2018.

Weibring, P., Richter, D., Walega, J. G., Rippe, L., and Fried, A.: Difference frequency generation spectrometer for simultaneous multispecies detection, Opt. Express, 18, 27670-27681, https://doi.org/10.1364/OE.18.027670, 2010.

Weinheimer, A. J., Walega, J. G., Ridley, B. A., Gary, B. L., Blake, D. R., Blake, N. J., Rowland, F. S., Sachse, G. W., Anderson, B. E., and Collins, J. E.: Meridional distributions of NOx, NOy and other species in the lower stratosphere and upper troposphere during AASE II, Geophys Res Lett, 21, 2583-2586, https://doi.org/10.1029/94gl01897, 1994.

Wisthaler, A., Hansel, A., Dickerson, R. R., and Crutzen, P. J.: Organic trace gas measurements by PTR-MS during INDOEX 1999, J. Geophys. Res. Atmos., 107, https://doi.org/10.1029/2001jd000576, 2002.

Wofsy, S. C., Afshar, S., Allen, H. M., Apel, E., Asher, E. C., Barletta, B., Bent, J., Bian, H., Biggs, B. C., Blake, D. R., Blake, N., Bourgeois, I., Brock, C. A., Brune, W. H., Budney, J. W., Bui, T. P., Butler, A., Campuzano-Jost, P., Chang, C. S., Chin, M., Commane, R., Correa, G., Crounse, J. D., Cullis, P. D., Daube, B. C., Day, D. A., Dean-Day, J. M., Dibb, J. E., DiGangi, J. P., Diskin, G. S., Dollner, M., Elkins, J. W., Erdesz, F., Fiore, A. M., Flynn, C. M., Froyd, K., Gesler, D. W., Hall, S. R., Hanisco, T. F., Hannun, R. A., Hills, A. J., Hintsa, E. J., Hoffman, A., Hornbrook, R. S., Huey, L. G., Hughes, S., Jimenez, J. L., Johnson, B. J., Katich, J. M., Keeling, R. F., Kim, M. J., Kupc, A., Lait, L. R., Lamarque, J.-F., Liu, J., McKain, K., Mclaughlin, R. J., Meinardi, S., Miller, D. O., Montzka, S. A., Moore, F. L., Morgan, E. J., Murphy, D. M., Murray, L. T., Nault, B. A., Neuman, J. A., Newman, P. A., Nicely, J. M., Pan, X., Paplawsky, W., Peischl, J., Prather, M. J., Price, D. J., Ray, E., Reeves, J. M., Richardson, M., Rollins, A. W., Rosenlof, K. H., Ryerson, T. B., Scheuer, E., Schill, G. P., Schroder, J. C., Schwarz, J. P., St.Clair, J. M., Steenrod, S. D., Stephens, B. B., Strode, S. A., Sweeney, C., Tanner, D., Teng, A. P., Thames, A. B., Thompson, C. R., Ullmann, K., Veres, P. R., Vieznor, N., Wagner, N. L., Watt, A., Weber, R., Weinzierl, B., Wennberg, P., Williamson, C. J., Wilson, J. C., Wolfe, G. M., Woods, C. T., and Zeng, L. H.: ATom: Merged Atmospheric Chemistry, Trace Gases, and Aerosols. ORNL DAAC, Oak Ridge, Tennessee, USA, https://doi.org/10.3334/ornldaac/1581, 2018.

Wooldridge, P. J., Perring, A. E., Bertram, T. H., Flocke, F. M., Roberts, J. M., Singh, H. B., Huey, L. G., Thornton, J. A., Wolfe, G. M., Murphy, J. G., Fry, J. L., Rollins, A. W., LaFranchi, B. W., and Cohen, R. C.: Total Peroxy Nitrates (ΣPNs) in the atmosphere: the Thermal Dissociation-Laser Induced Fluorescence (TD-LIF) technique and comparisons to speciated PAN measurements, Atmos. Meas. Tech., 3, 593-607, https://doi.org/10.5194/amt-3-593-2010, 2010.

Yacovitch, T. I., Herndon, S. C., Roscioli, J. R., Floerchinger, C., McGovern, R. M., Agnese, M., Petron, G., Kofler, J., Sweeney, C., Karion, A., Conley, S. A., Kort, E. A., Nahle, L., Fischer, M., Hildebrandt, L., Koeth, J., McManus, J. B., Nelson, D. D., Zahniser, M. S., and Kolb, C. E.: Demonstration of an ethane spectrometer for methane source identification, Environ. Sci. Technol., 48, 8028-8034, http://doi.org/10.1021/es501475q, 2014.

Zheng, W., Flocke, F. M., Tyndall, G. S., Swanson, A., Orlando, J. J., Roberts, J. M., Huey, L. G., and Tanner, D. J.: Characterization of a thermal decomposition chemical ionization mass spectrometer for the measurement of peroxy acyl nitrates (PANs) in the atmosphere, Atmos. Chem. Phys., 11, 6529-6547, https://doi.org/10.5194/acp-11-6529-2011, 2011.
